# Blood flukes (Digenea: Aporocotylidae) of marine fishes from off northern Patagonia, Argentina, with the redescription of *Aporocotyle argentinensis* Smith, 1969

**DOI:** 10.1016/j.crpvbd.2026.100399

**Published:** 2026-06-12

**Authors:** Marta Valmaseda-Angulo, Gema Alama-Bermejo, David I. Hernández-Mena, Francisco E. Montero, Jesús S. Hernández-Orts

**Affiliations:** a Marine Zoology Unit, Cavanilles Institute of Biodiversity and Evolutionary Biology, University of Valencia, Science Park, Paterna, Spain; b Department of Invertebrates, Natural History Museum of Geneva, Geneva, Switzerland; c Fish Health Division, Department for Farm Animals and Food System Science, University of Veterinary Medicine, Vienna, Austria; d Centro de Investigación Aplicada y Transferencia Tecnológica en Recursos Marinos Almirante Storni (CIMAS – CCT CONICET – CENPAT), San Antonio Oeste, Río Negro, Argentina; e Laboratorio de Helmintología, Instituto de Biología, Universidad Nacional Autónoma de México, Mexico City, Mexico; f Department of Life Sciences, Natural History Museum, London, SW7 5BD, UK; g Institute of Parasitology, Biology Centre, Czech Academy of Sciences, České Budějovice, Czech Republic

**Keywords:** Fish parasites, Molecular phylogeny, 28S rRNA gene, *cox*1, *Merluccius hubbsi*, *Genypterus blacodes*, SEM, CLSM, South America

## Abstract

*Aporocotyle* is the second richest genus of fish blood flukes within the family Aporocotylidae. These cosmopolitan trematodes infect the heart, bulbus arteriosus, and blood vessels of marine fishes belonging to the orders Gadiformes, Ophidiiformes, Perciformes, Pleuronectiformes, and Scorpaeniformes. In this study, three species of *Aporocotyle*, i.e. *A. argentinensis*, *A. mariachristinae*, and *A. ymakara*, from teleost fishes collected in the San Matías Gulf, Patagonia, Argentina, are morphologically and genetically characterized. Re-examination of these blood flukes provided new detailed drawings, measurements, photomicrographs, confocal and scanning electron microscopy (SEM) images. *Aporocotyle argentinensis* is redescribed based on type-, voucher- and newly collected material from the bulbus arteriosus, heart and gill blood vessels of the Argentine hake *Merluccius hubbsi* from Argentina. We generated partial sequences for the large subunit of the nuclear ribosomal RNA (28S rRNA) and the mitochondrial cytochrome *c* oxidase subunit 1 (*cox*1) genes for *A. argentinensis* and *A. ymakara* and used these sequences to investigate the phylogenetic relationships of these blood flukes. Our morphological analyses highlighted the taxonomic importance of the distribution of tegumental spines, the extension of the posterior caeca, and the presence of a genital atrium to circumscribe and classify species of *Aporocotyle*. Phylogenetic analyses recovered *A. mariachristinae* and *A. ymakara* as closely related taxa and placed the new sequences of *A. argentinensis* in a clade together with previously published sequences of isolates identified as *A. argentinensis* from three hake species (*Merluccius hubbsi*, *M. gayi* and *M. australis*) from Argentina, Chile, Peru and the Falkland Islands. This study provides a comprehensive review of the diversity of *Aporocotyle* in the southwest Atlantic, contributing to a better understanding of the systematics and evolutionary history of these blood flukes.

## Highlights

• Three blood fluke species (Digenea: Aporocotylidae) from Argentine marine fishes are reported.
• *Aporocotyle argentinensis* is redescribed in the light of the new data.
• Tegumental spines, posterior caeca and genital atrium delimit *Aporocotyle* spp.
• Confocal laser scanning microscopy facilitates visualization of spine clusters.
• Phylogenetic relationships of these blood flukes are investigated using 28S rRNA and *cox*1 genes.

## Graphical abstract

**Figure:**
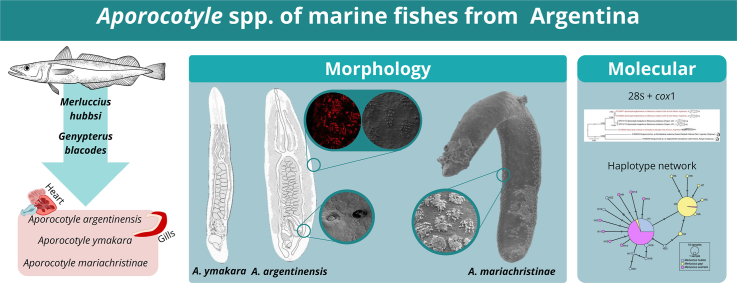

## 1 Introduction

The blood fluke fauna of marine fishes from the southwestern Atlantic off Argentina has largely been neglected. Despite sustained research efforts in this country over the last three decades to address gaps in marine fish parasitology, studies focusing on the taxonomy and systematics of fish blood flukes remain notably scarce. To the best of our knowledge, four species belonging to the family Aporocotylidae Odhner, 1912, have been reported from marine fishes off Argentina: *Aporocotyle argentinensis* Smith, 1969; *A. mariachristinae* Hernández-Orts, Alama-Bermejo, Carrillo, García, Crespo, Raga & Montero, 2012; *A. ymakara* Villalba & Fernández, 1986 ; and *Cardicola ambrosioi* Braicovich, Etchegoin, Timi & Sardella, 2006 (Smith, 1969; Braicovich et al., 2006; Hernández-Orts et al., 2012). Species of the Aporocotylidae are richest in the more derived groups of marine and estuarine ray-finned fishes (Euteleostei) (Warren and Bullard, 2023). The life cycles of most blood fluke species are unknown, but available evidence indicates that the asexual reproduction occurs in the body cavity of estuarine and marine polychaetes, and adults infect the lymphatic and blood vascular system of fishes (Warren and Bullard, 2023). The life cycle of aporocotylids lacks both a metacercarial stage and a second intermediate host, unlike the typical three-host trematode life cycle.

*Aporocotyle* Odhner, 1900, the type-genus of the Aporocotylidae, comprises 18 species that parasitize marine fishes in the Atlantic, Pacific, and Indian Oceans, as well as the Ross Sea in Antarctica (Santoro et al., 2015). Nearly half of the species (8 spp.) of *Aporocotyle* are reported along the Pacific and Atlantic coasts of South America (see table 1 in Hernández-Orts et al., 2012). Of these, one species has been originally described from off Peru, i.e. *A. garciai* Tantaleán & Martínez, 1990 from an unidentified species of *Genypterus* (Ophidiiformes) (Tantaleán and Martínez, 1990); five from off Chile, i.e. *A. australis* Fernández & Durán, 1985 from the Southern hake *Merluccius australis* (Hutton) (Merlucciidae), *A. keli* Villalba & Fernández, 1986 from the red cusk-eel *Genypterus chilensis* Guichenot, *A. kuri* Villalba & Fernández, 1986 from the black cusk-eel *Genypterus maculatus* (Tschudi), *A. wilhelmi* Villalba & Fernández, 1986 from the South Pacific hake *Merluccius gayi* (Guichenot) and *A. ymakara* Villalba & Fernández, 1986 from the pink cusk-eel *Genypterus blacodes* (Förster) (Fernández and Durán, 1985; Villalba and Fernández, 1986a, 1986b); and three from off Argentina, i.e. *A. argentinensis* from the Argentine hake *Merluccius hubbsi* Marini, *A. mariachristinae* and *A. ymakara* from the pink cusk-eel *G. blacodes* (Smith, 1969; Hernández-Orts et al., 2012). Since their original descriptions, most of these species of *Aporocotyle* have subsequently been reported from various localities along the Pacific and Atlantic coasts of South America (MacKenzie and Longshaw, 1995; George-Nascimento, 1996; Sardella and Timi, 1996; Oliva and Ballón, 2002; Martínez et al., 2013; Chero et al., 2014; González-Poblete et al., 2022; Oliva et al., 2022) or even the Pacific coast off central Japan (Kamegai et al., 2002).

The taxonomic status of the species of *Aporocotyle* from South American waters has been questioned in recent years. This is partly because molecular data are currently available in GenBank for only two nominal species, *A. argentinensis* and *A. mariachristinae*, and because the morphological and ecological characters used to distinguish them are subtle (Villalba and Fernández, 1986b; Hernández-Orts et al., 2012). An extensive genetic survey using ribosomal and mitochondrial genes for aporocotylids recovered from three hake species (*M. hubbsi*, *M. australis*, and *M. gayi*) from the Pacific (off Chile and Peru) and Atlantic (off Argentina and the Falkland Islands) coasts of South America was conducted by Oliva et al. (2022). These three hake species are the reported hosts for *A. argentinensis*, *A. australis*, and *A. wilhelmi*, respectively (see above). However, Oliva et al. (2022) detected minimal genetic diversity and therefore concluded that these hakes from off South America are infected by a single species of *Aporocotyle*, suggesting that “*A. australis* and *A. wilhelmi* should be considered as junior synonyms of *A. argentinensis*”. In addition, Oliva et al. (2022) did not link their molecular findings with a comprehensive morphological dataset compiled from both newly collected and museum material. Therefore, the taxonomic action proposed by Oliva et al. (2022) remains controversial and should be revised.

This study presents the results of morphological and molecular phylogenetic analyses of three species of *Aporocotyle* from marine fishes off northern Patagonia, Argentina. We provide a redescription of *A. argentinensis* based on the re-examination of type-, voucher-, and newly collected material from Argentine hakes *M. hubbsi*. New morphological data are provided for *A. ymakara*, confocal photomicrographs for *A. argentinensis*, and scanning electron photomicrographs for *A. argentinensis* and *A. mariachristinae* are presented for the first time. In addition, *A. argentinensis* and *A. ymakara* are characterized molecularly using the large subunit of the nuclear ribosomal RNA (28S rRNA) and the mitochondrial cytochrome *c* oxidase subunit 1 (*cox*1) genes. These newly generated sequences were used to explore phylogenetic relationships within *Aporocotyle*. Finally, we generated a haplotype network based on *cox*1 sequences of *A. argentinensis* from hakes collected off Argentina, Chile, Peru, and the Falkland Islands to assess the genetic diversity of this blood fluke species.

## 2 Materials and methods

### 2.1 Sample collection

Five Argentine hakes *M. hubbsi* (total body length: 46–61 cm) and nine pink cusk-eels *G. blacodes* (total body length: 51–81 cm) were purchased from local fishermen at San Antonio Oeste, Río Negro, Argentina, between March 2017 and June 2019. Fishes were caught in San Matías Gulf basin (41–42°S, 63–65°W) by commercial bottom trawling vessels and kept fresh on ice. The heart, bulbus arteriosus, ventral aorta and gills were excised from the fishes, placed in Petri dishes with saline solution and examined using a dissecting microscope. Blood flukes were gently washed in saline and fixed in hot 4% formaldehyde solution or in 96% ethanol. Hologenophores were prepared of five blood fluke specimens from Argentine hakes *M. hubbsi* and one from a pink cusk-eel *G. blacodes*, following Pleijel et al. (2018). Small pieces of tissue were excised from the anterior portion of the body and used for DNA isolation and sequencing, with the remaining part retained as a morphological voucher and fixed in 4% formaldehyde.

### 2.2 Morphological data

Blood flukes were stained with Mayer’s hydrochloric carmine solution, destained in 70% ethanol and HCl, dehydrated in an ethanol series, cleared with methyl salicylate and mounted in Canada balsam. Specimens were examined with an Olympus BX51 and a Leica DM5000B compound microscope. Measurements were taken from drawings made with the aid of a drawing tube. Illustrations were prepared following Villora-Montero et al. (2025). Blood flukes were identified according to Smith (1969), Villalba and Fernández (1986b), and Hernández-Orts et al. (2012).

Fish blood flukes collected in the present study were compared with the type- and voucher specimens of *A. argentinensis* deposited at the Parasitic Worms collection of the Natural History Museum, London, UK (NHMUK): holotype (NHMUK, 1969.5.15.1), and two vouchers (NHMUK, 1994.6.7.30–34). Vouchers from this study are deposited in the Helminthological Collection of the Museo de La Plata, Buenos Aires, Argentina (HCMLP-He), the Natural History Museum, Geneva, Switzerland (MHNG-PLAT), the Colección Nacional de Helmintos, Mexico City, Mexico (CNHE), and the NHM, London, UK.

The following abbreviations for the metrical features were used in the tables: BL, body length; BW, maximum body width; BCL, buccal capsule length; BCW, buccal capsule width; SL, spine length; NSC, number of spines per cluster; OL, oesophagus length; MEPC, maximum extension of posterior caeca; RACL, right anterior caecum length; LACL, left anterior caecum length; RPCL, right posterior caecum length; LPCL, left posterior caecum length; NT, number of testes; TLA, testis long axis; TWA, testis wide axis; CSL, cirrus-sac length; CSW, cirrus-sac width; CSWW, cirrus-sac wall width; DPGP, distance from posterior body end to genital pore; OVL, ovary length; OVW, ovary width; MGL, Mehlis’ gland length; MGW, Mehlis’ gland width; USRL, uterine seminal receptacle length, ML, metraterm length; EL, egg-length; EW, egg-width; DANC, distance from anterior body end to nerve commissure.

In addition, the following ratios were calculated: OL/BL, oesophagus to body length ratio; RACL/RPCL, right anterior caeca to right posterior caeca length ratio; LACL/LPCL, left anterior caeca to left posterior caeca length ratio; RPCL/BL, right posterior caeca to body length ratio; LPCL/BL, left posterior caeca to body length ratio.

Two mounted specimens of *A. argentinensis* were examined using an Olympus FV1000 confocal laser scanning microscope. To visualize the autofluorescence of sclerotized structures, a 559 nm excitation laser line was employed. Optical sections were acquired to produce a Z-stack consisting of 49 slices with a 450 nm step size. The resulting images were processed and analyzed using Fiji (Schindelin et al., 2012) to generate maximum intensity projections and evaluate the three-dimensional architecture of the spines.

Four blood flukes from Argentine hakes *M. hubbsi* fixed in 4% formaldehyde solution were studied by scanning electron microscopy (SEM). Worms were post-fixed in 2% osmium tetroxide for 2 h, washed with 0.1 M phosphate-buffered saline (pH 7.4), dehydrated in an ethanol series, mounted on aluminium stubs using double-sided carbon tape, sputter coated with 30-nm gold/palladium, and examined with a Jeol JSEM 7401F microscope (JEOL Ltd., Tokyo, Japan) at the Laboratory of Electron Microscopy, Biology Centre, Czech Academy of Sciences, at an accelerating voltage of 4 kV. Two blood flukes from pink cusk-eels *G. blacodes* were prepared for SEM following Hernández-Mena et al. (2016) and examined with a Hitachi Stereoscan Model S-2469N scanning electron microscope, operating at 15 kV at the Instituto de Biología, Universidad Nacional Autónoma de México (UNAM).

### 2.3 DNA extraction, PCR and phylogenetic analyses

Small pieces from the anterior part of selected blood flukes were air-dried and then added to TNES prior to DNA extraction using phenol-chloroform. Polymerase chain reaction (PCR) amplifications were carried out in 25 μl reactions, containing 12.5 μl of AccuStart II PCR SuperMix (Quanta Bio, Beverly, USA), 8 μl of RNAse/DNAse-free water, 1.5 μl of each primer (10 mM) and 1.5 μl of DNA.

Partial fragments of the 28S gene (domains D1-D3; ∼1400 bp) were amplified using the forward primer 391 (5′-AGC GGA GGA AAA GAA ACT AA-3′) (Nadler and Hudspeth, 1998) and the reverse primer 536 (5′-CAG CTA TCC TGA GGG AAA C-3′) (Stock et al., 2001); amplification profile: 5 min at 94 °C, followed by 35 cycles of 94 °C for 1 min, 55 °C for 1 min, 72 °C for 1 min, and a final extension step of 72 °C for 10 min. A ∼630-bp region near the 5′ end (barcode or Folmer region) of the *cox*1 gene was amplified using the forward primer DICE1F (5′-ATT AAC CCT CAC TAA ATT WCN TTR GAT CAT AAG-3′) (Moszczynska et al., 2009) and the reverse primer DICE14R (5′-TAA TAC GAC TCA CTA TAC CHA CMR TAA ACA TAT GAT G-3′) (Van Steenkiste et al., 2015); amplification profile: 4 min at 94 °C, followed by 40 cycles of 94 °C for 40 s, 53 °C for 30 s, 72 °C for 1 min, and final extension step of 72 °C for 7 min. Finally, a ∼900-bp fragment of the middle region of *cox*1 gene was amplified using the forward primer JB3 (5′-TTT TTT GGG CAT CCT GAC GTT TAT-3′) and the reverse primer CO1-R trema (5′-CAA CAA ATC ATG ATG CAA AAG G-3′) (Miura et al., 2005); amplification profile: 1 min at 94 °C, followed by 35 cycles of 94 °C for 30 s, 53 °C for 30 s, 72 °C for 1 min, and a final extension step of 72 °C for 7 min.

PCR products were purified before sequencing using ExoSAP-IT™ (GE Healthcare Life Sciences, Buckinghamshire, UK) following the manufacturer’s instructions. Sanger sequencing was performed by SEQme (Dobříš, Czech Republic), using the PCR primers and the additional internal primers 504 (5′-CGT CTT GAA ACA CGG ACT AAGG-3′) and 503 (5′-CCT TGG TCC GTG TTT CAA GAC G-3′) (Stock et al., 2001) for 28S rRNA gene. Contiguous sequences were assembled and edited using Geneious Prime v.2025.1.3 (https://www.geneious.com) and deposited in the GenBank database under the accession numbers PZ168269-PZ168273, PZ168637-PZ168639, and PZ168640-PZ168644.

Phylogenetic analyses were only performed for 28S rRNA gene and the barcode region of *cox*1, as sequences for the middle region of *cox*1 are only available for *A. argentinensis* on GenBank (Oliva et al., 2022; see below). Newly generated sequences were aligned together with sequences of five species of *Aporocotyle* retrieved from GenBank (Table 1) using MAFFT v. 7.490 (Katoh and Standley, 2013) under default parameters implemented in Geneious Prime. *Paradeontacylix iberica* Repullés-Albelda, Montero, Holzer, Ogawa, Hutson & Raga, 2008 (GenBank: AM489593) was used to root the tree of the 28S rDNA dataset, and Sanguinicolidae gen. sp. (GenBank: AY829239, AY829240) was used to root the tree of the *cox*1 dataset. Potential mutational saturation and base composition bias were assessed for all codon positions of the *cox*1 dataset. Substitution saturation was examined using the substitution saturation test function *sensu* Xia et al. (2003) and Xia and Lemey (2009) and implemented in DAMBE v.7.2 (Xia, 2018). The alignment showed no significant substitution saturation (*Iss* = 0.585 < *Iss.c* = 0.775 and 0.684 for symmetrical and asymmetrical topologies, respectively; *P* < 0.01). To test for non-stationarity among taxa, a *χ*^2^ test of base frequency homogeneity was performed in PAUP v.4.0 (Swofford, 2002). The analysis confirmed nucleotide composition homogeneity across all evaluated taxa (*χ*^2^ = 12.63, *df* = 18, *P* = 0.813). Consequently, the complete *cox*1 dataset was used in all subsequent phylogenetic analyses.

**Table 1:** Taxa included in the phylogenetic analyses with data on the host, locality and GenBank accession numbers (28S rDNA and *cox*1).

Species	Host	Locality	GenBank ID		Source
			28S rDNA	*cox*1^a^	
*Aporocotyle argentinensis* Smith, 1969	*Merluccius australis* (Hutton)	Off Puerto Montt, Chile	KY491743-KY491745	–	Oliva et al. (2022)
		Off Puerto Madryn, Argentina	KY491754-KY491756	–	
	*Merluccius gayi* (Guichenot)	Off Callao, Peru	KY492000-KY492002	–	
		Off Coquimbo, Chile	KY492005-KY492007	–	
		Off Constitución, Chile	KY492017-KY492019	–	
		Off Talcahuano, Chile	KY492025-KY492027	–	
	*Merluccius hubbsi* Marini	Off Mar del Plata, Argentina	KY492037	–	
		Off Falkland Islands, UK	KY492038, KY492039	–	
		Off Patagonia, Argentina	JX094803	–	Hernández-Orts et al. (2012)
		Off Golfo de San Matías, Argentina	PZ168269-PZ168272	PZ168637-PZ168638	Present study
*Aporocotyle margolisi* Smith, 1967	*Merluccius productus* (Ayres)	Off Oregon, USA	MF287915, MF287916	MF314114, MF314115	Hernández-Orts et al. (2017)
*Aporocotyle mariachristinae* Hernández-Orts, Alama-Bermejo, Carrillo, García, Crespo, Raga & Montero, 2012	*Genypterus blacodes* (Forster)	Off Patagonia, Argentina	JX094802	–	Hernández-Orts et al. (2012)
*Aporocotyle michaudi* Santoro, Cipriani, Pankov & Lawton, 2015	*Trematomus bernacchii* Boulenger	Ross Sea, Antarctica	KR025807	–	Santoro et al. (2015)
*Aporocotyle spinosicanalis* Williams, 1958	*Merluccius merluccius* (L.)	North Sea	AF167094	–	Snyder and Loker (2000)
		Off UK	AY222177	–	Olson et al. (2003)
*Aporocotyle ymakara* Villalba & Fernández, 1986	*G. blacodes*	San Matias Gulf, off Argentina	PZ168273	PZ168639	Present study
Outgroup					
*Paradeontacylix iberica* Repullés-Albelda, Montero, Holzer, Ogawa, Hutson & Raga, 2008	*Seriola dumerili* (Risso)	Off Murcia, Spain	AM489593	–	Repullés-Albelda et al. (2008)
Sanguinicolidae gen. sp.	*Biomphalaria sudanica*	Queen Elizabeth National Park, Uganda	–	AY829239	Brant et al. (2006)
	*Segmentorbis kanisaensis*	Kisumu, Kenya	–	AY829240	Brant et al. (2006)

a Barcode region.

Phylogenetic relationships of *Aporocotyle* spp. were reconstructed under Bayesian inference (BI) and maximum likelihood (ML) criteria. The best-fit nucleotide substitution model for each gene dataset was determined using ModelFinder (Kalyaanamoorthy et al., 2017) implemented in IQ-TREE v.3.0.1 (Wong et al., 2026) according to the corrected Akaike information criterion. These were the Hasegawa-Kishino-Yano model with estimates of invariant sites (HKY+I) for the 28S rDNA dataset and the general time-reversible model with gamma-distributed rate variation among sites (GTR+G) for the *cox*1 barcode region dataset. The BI analyses were performed using MrBayes v.3.2.7 (Ronquist et al., 2012), and the posterior probabilities were calculated *via* two independent Markov Chain Monte Carlo runs of four chains with standard settings for 20,000,000 generations with a sampling frequency of 1000 generations. The “burn-in” was defined as the point at which the average S.D. of split frequencies was < 0.01. ML analyses were run in IQ-TREE, and 1000 standard bootstrap replicates were run to assess nodal support. Trees were visualized using FigTree v.1.4.4. (Rambaut, 2009). Pairwise genetic distances (uncorrected p-distance) were calculated with MEGA 12 (Kumar et al., 2024), using the gaps/missing data treatment as pairwise deletion.

A haplotype network for *A. argentinensis* was constructed using novel and published sequences for the middle region of *cox*1 (Table 2). Sequences were aligned as described above, and the extremes of the alignment were trimmed to match the shortest sequence. An unrooted statistical parsimony haplotype network was constructed using DNAsp6 v.6.12.03 (Rozas et al., 2017) to infer the number of haplotypes from DNA sequences and to calculate the number of polymorphic sites (*κ*), haplotype diversity (*Hd*) and nucleotide diversity (*π*). Finally, the relationships among haplotypes were assessed with a haplotype network constructed using the median-joining method in PopART v.1.7 (Bandelt et al., 1999).

**Table 2:** Partial sequences of the middle region of *cox*1 of *Aporocotyle argentinensis* ex *Merluccius* spp. included in the haplotype analysis with data on the host, locality and GenBank accession numbers.

Host species	Locality	GenBank ID
*M*. *gayi*	Callao, Peru	KY491915-KY491919^a^
*M. gayi*	Coquimbo, Chile	KY491900-KY491914^a^
*M. gayi*	Constitución, Chile	KY491959-KY491966^a^
*M. gayi*	Talcahuano, Chile	KY491937-KY491958^a^
*M. gayi*	Duao, Chile	KY491920-KY491936^a^
*M. australis*	Puerto Montt, Chile	KY491831-KY491861^a^
*M. australis*	Guaitecas, Chile	KY491824-KY491830^a^
*M. australis*	Puerto Aysen, Chile	KY491885-KY491899^a^
*M. australis*	Puerto Madryn, Argentina	KY491862-KY491884^a^
*M*. *hubbsi*	Mar del Plata, Argentina	OK338724-OK338754^a^
*M. hubbsi*	Golfo de San Matías, Argentina	PZ168640-PZ168644^b^

a Source: Oliva et al. (2022).

b Source: Present study.

## 3 Results

### 3.1 *Aporocotyle argentinensis* Smith, 1969

#### 3.1.1 Taxonomic summary

*Type-host (same host in this study)*: Argentine hake *Merluccius hubbsi* Marini (Gadiformes: Merlucciidae).

*Other hosts*: Southern hake *Merluccius australis* (Hutton) (Gadiformes: Merlucciidae); South Pacific hake *Merluccius gayi* (Guichenot) (Gadiformes: Merlucciidae).

*Type-locality*: Coast of Argentina, southwestern Atlantic.

*Locality (this study)*: San Matías Gulf (an inlet of the Atlantic Ocean, 40°42′–40°45′S, 63°05′–65°10′W), off Patagonia, Argentina (collected on 14–16.v.2017).

*Other localities*: *Merluccius hubbsi*: off Falkland Islands, UK (MacKenzie and Longshaw, 1995; Oliva et al., 2022); Argentinian-Uruguayan common fishing zone (Sardella and Timi, 1996); off North (42°45′S–42°59′S, 61°09′W–62°58′W) and central Patagonia (47°00′S–47°19′S, 61°59′W–64°25′W), Argentina (Hernández-Orts et al., 2012); off Mar del Plata, Argentina (Oliva et al., 2022). *Merluccius gayi*: off Callao, Peru (Oliva et al., 2022); off Coquimbo, Duao, Constitución and Talcahuano, Chile (Oliva et al., 2022). *Merluccius australis*: off Puerto Madryn, Argentina (Oliva et al., 2022); off Falkland Islands, UK (Oliva et al., 2022); off Puerto Montt and Guaitecas Island, Chile (Oliva et al., 2022).

*Site in host*: Bulbus arteriosus, heart and gill blood vessels.

*Material studied*: Type-material: ex *M. hubbsi,* bulbus arteriosus, off Argentinian coast, holotype NHMUK 1969.5.15.1. Comparative material: ex *M. hubbsi*, heart and blood vessels, off Falkland Islands/Islas Malvinas, NHMUK 1994.6.7.30-34 (2 specimens). New material: ex *M. hubbsi*, bulbus arteriosus and heart, San Matías Gulf, Patagonia, Argentina, 25 voucher specimens: HCMLP-He 8349 (3 specimens), MHNG-PLAT-0160159 (2 specimens), CNHE 11087 (3 specimens) and NHMUK 2025.10.31.1-17 (17 specimens). ex *M. hubbsi*, gill blood vessels, San Matías Gulf, Patagonia, Argentina, 13 voucher specimens: HCMLP-He 8350 (5 specimens), MHNG-PLAT-0160182 (3 specimens), CNHE 11086 (5 specimens).

*Representative DNA sequences*: PZ168269-PZ168272 (28S rDNA), PZ168637-PZ168638 (*cox*1 barcode region), and PZ168640-PZ168644 (*cox*1 middle region).

*Prevalence (this study)*: Bulbus arteriosus and heart: 60% (*n* = 5); gill blood vessels: 100% (*n* = 5).

*Mean abundance (this study)*: Bulbus arteriosus and heart: 9 worms per fish; gill blood vessels: 45.6 worms per fish.

*Mean intensity (this study)*: Bulbus arteriosus and heart: 9 worms per infected fish; gill blood vessels: 45.6 worms per infected fish.

*Intensity range (this study)*: Bulbus arteriosus and heart: 8–10 worms; gill blood vessels: 10–80 worms.

#### 3.1.2 Description

**Fig. 1:**
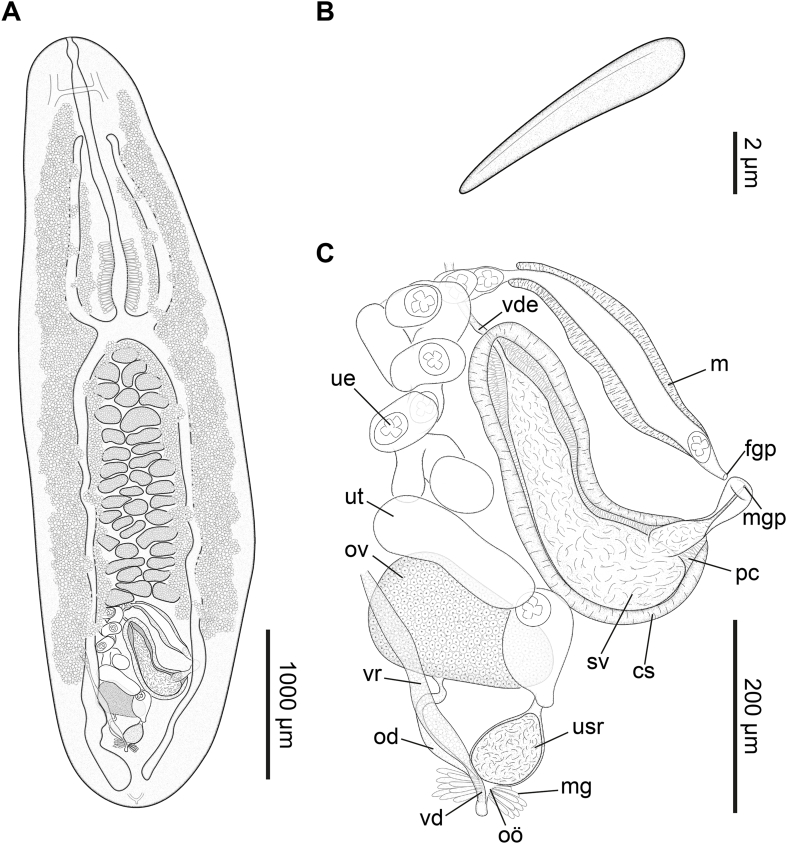
*Aporocotyle argentinensis* Smith, 1969 ex *Merluccius hubbsi* Marini. **A** Whole worm, ventral view. **B** Tegumental spine, ventral view. **C** Reproductive organs, ventral view. *Abbreviations*: cs, cirrus-sac; fgp, female genital pore; m, metraterm; mg, Mehlis’ gland; mgp, male genital pore; od, oviduct; oö, oötype; ov, ovary; pc, prostatic cells; sv, seminal vesicle; ue, uterine egg; usr, uterine seminal receptacle; ut, uterus; vde, vas deferens; vd, vitelloduct; vr, vitelline reservoir.

**Fig. 2:**
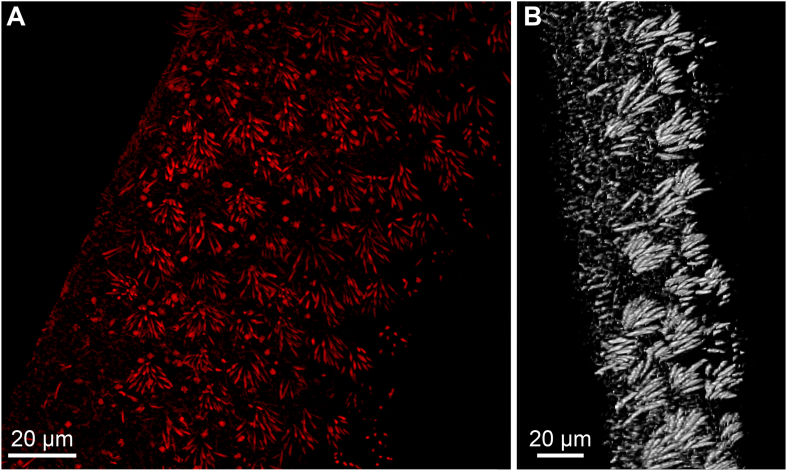
Confocal laser scanning microscope images of *Aporocotyle argentinensis* Smith, 1969 ex *Merluccius hubbsi* Marini showing the general arrangement of the clusters of spines at the level of intestinal bifurcation. **A** Z-stack of clusters. **B** Three-dimensional reconstruction of the clusters of spines.

**Fig. 3:**
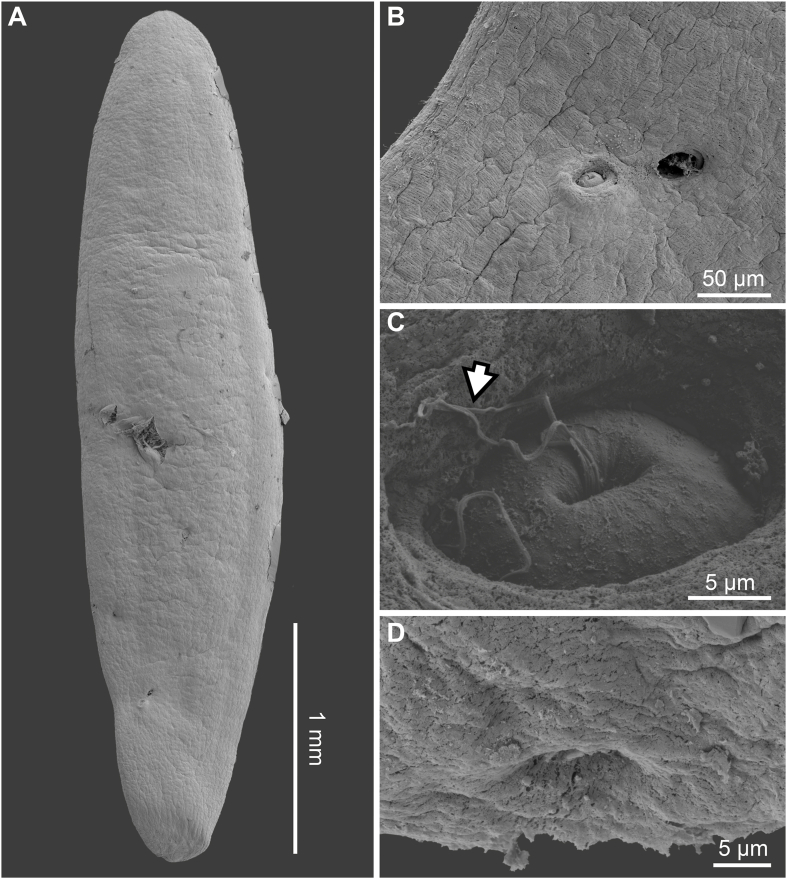
Scanning electron microscope images of *Aporocotyle argentinensis* Smith, 1969 ex *Merluccius hubbsi* Marini. **A** Whole worm, dorsal view. **B** Male and female genital pores, dorsal view. **C** Detail of the male genital pore with spermatozoa (*arrow*), dorsal view. **D** Excretory pore, dorsal view.

**Fig. 4:**
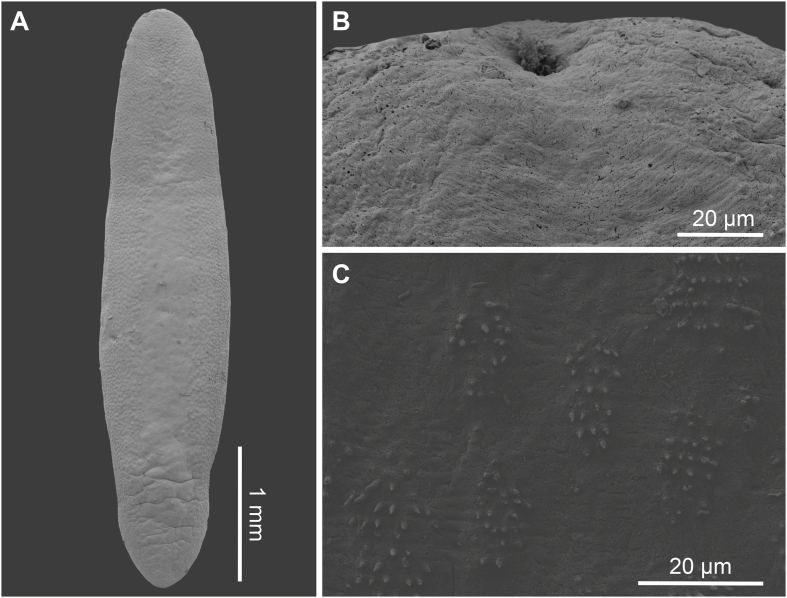
Scanning electron microscope images of *Aporocotyle argentinensis* Smith, 1969 ex *Merluccius hubbsi* Marini. **A** Whole worm, ventral view. **B** Mouth, ventral view. **C** Details of clusters of spines, ventral view.

**Fig. 5:**
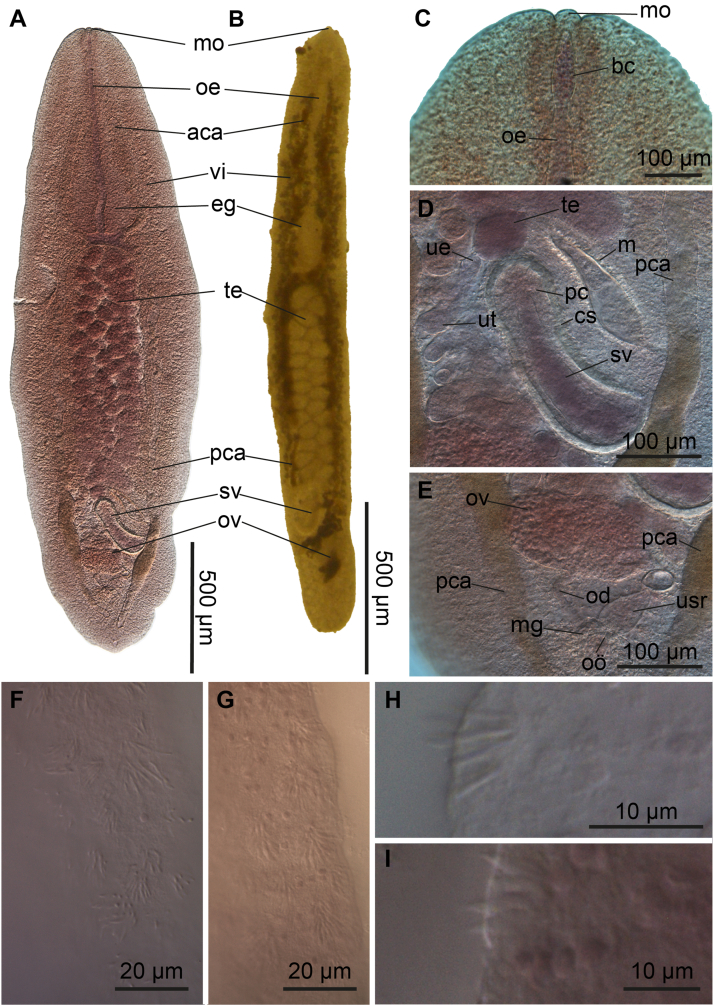
Light microscope photomicrographs of *Aporocotyle* spp. from marine fishes in San Matías Gulf, off Patagonia, Argentina. *Aporocotyle argentinensis* Smith, 1969 ex *Merluccius hubbsi* Marini: **A** Stained whole worm, ventral view. **C** Anterior extremity of the worm, ventral view. **D** Cirrus-sac and metraterm, ventral view. **E** Distal part of the female reproductive system. **F** Ventro-lateral body area showing the distribution of clusters of spines. **H** Tegumental spines, lateral view. *Aporocotyle ymakara* Villalba & Fernández, 1986 (hologenophore) ex *Genypterus blacodes* (Förster): **B** Fresh whole worm, ventral view. **G** Ventro-lateral body area showing the distribution of clusters of spines. **I** Clusters of spines. *Abbreviations*: aca, anterior caecum; bc, buccal capsule; cs, cirrus-sac; eg, oesophageal glands; m, metraterm; mg, Mehlis’ gland; mo, mouth; oe, oesophagus; od, oviduct; oö, oötype; ov, ovary; pc, prostatic cells; pca, posterior caeca; sv, seminal vesicle; te, testis; ue, uterine egg; usr, uterine seminal receptacle; ut, uterus; vi, vitellarium.

**Fig. 6:**
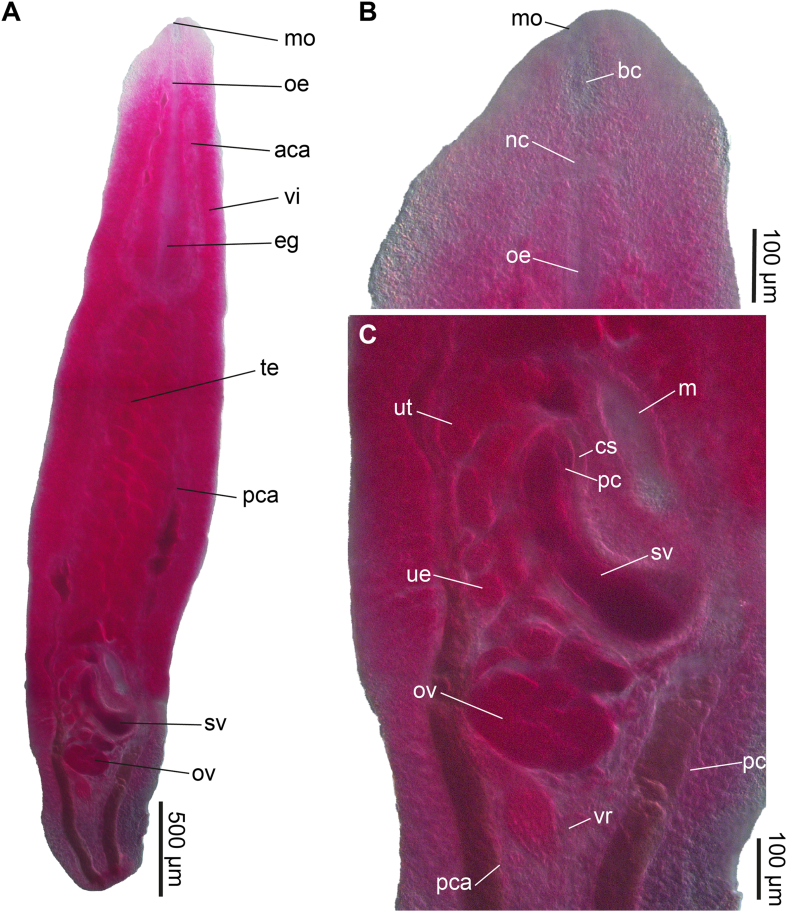
Holotype (NHMUK, 1969.5.15.1) of *Aporocotyle argentinensis* Smith, 1969 ex *Merluccius hubbsi* Marini, ventral view. **A** Whole worm. **B** Anterior region. **C** Reproductive organs. *Abbreviations*: aca, anterior caecum; bc, buccal capsule; cs, cirrus-sac; eg, oesophageal glands; m, metraterm; mo, mouth; nc, nerve commissure; oe, oesophagus; ov, ovary; pc, prostatic cells; pca, posterior caeca; sv, seminal vesicle; te, testis; ue, uterine egg; ut, uterus; vi, vitellarium; vr, vitelline reservoir.

[Based on type- and comparative material; morphometric data in Table 3; Fig. 1, Fig. 2, Fig. 3, Fig. 4, Fig. 5, Fig. 6.] Body elongate, with blunt anterior end and slightly pointed posterior end (Fig. 1A, 3A and 4A, 5A); maximum width approximately at midbody. Tegumental spines short, pointed (Fig. 1B and 2A, B, 5H), forming clusters of 17 to 40 spines each (Fig. 2A, B, 4C, 5F, H); ventral clusters of spines distributed along ventro-lateral margins of body, joining at sagittal axis from posterior end of mouth to approximately level of intestinal bifurcation (Fig. 4A); dorsal clusters arranged along dorso-lateral body margins, never joining at sagittal axis; clusters of spines less abundant at posterior half body (Fig. 3A). Oral sucker absent. Mouth subterminal (Fig. 1A and 4B), leading to an oval, spineless buccal capsule (Fig. 5C). Oesophagus long, surrounded by glands from its posterior third quarter to caeca bifurcation (Fig. 1A). Intestine H-shaped (Fig. 1A and 5A). Anterior caeca similar in length, extending anteriorly to approximately first third of oesophagus. Posterior caeca similar in length, extending to almost posterior body end (Fig. 1A and 5A).

**Table 3:** Comparative measurements of *Aporocotyle* spp. in *Merluccius* spp. from the Southeast Pacific and Southwest Atlantic. Measurements are in micrometres unless otherwise indicated.

Source	Smith (1969); Present study (NHMUK, 1969.5.15.1)	Mackenzie and Longshaw (1995); Present study (NHMUK, 1994.6.7.30-34)	Present study		Fernández and Durán (1985)	Villalba and Fernández (1986a)
Identified as	*A. argentinensis*	*A. argentinensis*	*A. argentinensis*		*A. australis*	*A. wilhelmi*
Host	*M. hubbsi*	*M. hubbsi*	*M. hubbsi*		*M. australis*	*M. gayi*
Locality	Atlantic coast of Argentina	Falkland Islands, UK	San Matías Gulf, Argentina		Guafo Island, Chile	Concepción Bay, Chile
Site in host	Heart	Heart	Gills	Heart	Gills and heart	Gills and heart
Feature	Range (*n* = 4)	Range (*n* = 2)	Range (*n* = 20)	Range (*n* = 10)	Range (*n* = 28)	Range (*n* = 25)
BL × BW (mm)	4.1–4.7 × 0.9–1.0 (4.7 × 0.9)^a^	4.5–4.7 × 0.8–0.9^c^	2.5–3.9 × 0.6–1.0	2.2–3.8 × 0.7–1.0	5.4–8.0 × 1.2–1.9	2.6–5.1 × 0.56–1.31
BCL × BCW	– (116 × 50)^a^	59–91 × 22–41^c^	44–100 × 22–59	65–93 × 35–68	–	90 × 30
SL	17	–	7–9	16–20	10	7–10
NSC	20–40 (−)	–	17–26	24–31	20–30	–
OL (mm)	1.3–1.5 (1.4)^a^	1.2–1.4^c^	0.8–1.4	0.7–1.2	1.5–2.2	0.9–1.7
MEPC	Posterior body end^b^	Posterior body end^c^	Posterior body end	Posterior body end	Posterior body end	Posterior body end
RACL	– (1078)^a^	1013–1130^c^	610–1143	687–1005	1215^b^	956^b^
LACL	– (1000)^a^	883–1065^c^	636–987	701–1060	1044^b^	917^b^
RPCL	– (3312)^a^	3091–3273^c^	1390–2442	2038–3005	4512^b^	2408^b^
LPCL	– (3221)^a^	3195–3247^c^	1364–2351	2047–3031	4417^b^	2489^b^
NT	– (39)^a^	35–41^c^	35–41	40–42	56–71	33–40
TLA × TWA	– (72–121 × 131–156)^a^	88–188^a^ × 78–122^c^	58–102 × 35–68	99–192 × 51–109	144–235 × 100–162^b^	121–170 × 62–120^b^
CSL × CSW	440–600 × 110–150 (447 × 156)^a^	338–384 × 94–128^c^	338–384 × 94–128	325–521 × 80–105	350–530 × 110–150	250–460 × 90–170
CSWW	– (19)^a^	9–31^c^	9–28	12–16	–	–
DPGP	650–1030 (494)^a^	822–856^c^	822–856	480–850	1128^b^	767^b^
OVL × OVW	230–270 × 140–170 (175 × 247)^a^	103 × 184^c^	66–153 × 66–153	72–139 × 125–235	160–200 × 280–400	87–160 × 130–290
MGL × MGW	– (50 × 72)^a^	34–44 × 53–72^c^	28–72 × 66–144	79–141 × 99–149	100 × 83^b^	–
USRL	– (97)^a^	66–75^c^	63–100	56–106	246^b^	–
ML	– (97)^a^	275–322^c^	197–375	213–337	530^b^	–
EL × EW	40 × 20 (48 × 48)^a^	34–50 × 27–38^c^	22–47 × 23–39	28–53 × 25–45	–	–
DANC	– (206)^a^	134–144^c^	94–194	131–179	–	–
*Ratios*						
OL/BL	– (1:3.4)^a^	1:3.3–3.6^c^	1:2.6–3.2	1:3.0–3.5	1:3.7^b^	1:3.0^b^
RACL/RPCL	– (1:3.1)^a^	1:2.7–3.2^c^	1:1.6–2.8	1:2.6–3.6	1:3.6^b^	1:2.5^b^
LACL/LPCL	– (1:3.2)^a^	1:3.0–3.7^c^	1:1.7–2.8	1:2.6–3.1	1:4.1^b^	1:2.7^b^
RPCL/BL	– (1:1.4)^a^	1:1.4–1.5^c^	1:1.6–1.9	1:1.3–1.4	1:1.4^b^	1:1.5^b^
LPCL/BL	– (1:1.5)^a^	1:1.4–1.5^c^	1:1.5–1.8	1:1.3–1.4	1:1.4^b^	1:1.5^b^

a Metrical and morphological observations obtained from re-examination of the holotype in parentheses.

b Obtained from the published figure.

c Metrical and morphological observations obtained from voucher material.

Testes irregularly to transversely-oval, intercaecal, between intestinal bifurcation and rest of genitalia (Fig. 1A and 5A). Vas deferens extending from posterior margin of testicular field. Cirrus-sac claviform, with thick muscular wall, slightly constricted in the middle, directing sinistrally, ending in male genital pore, contains elongate and slightly sinuous seminal vesicle surrounded by numerous prostatic cells (Fig. 1C and 5D). Cirrus not observed. Male and female genital pores dorso-sinistral, intercaecal, at about 24–26% of body length from posterior body margin (Fig. 3B). Spermatozoa released through male genital pore observed (Fig. 3C).

Ovary slightly dextral, intercaecal, transversely-oval to subtriangular (Fig. 1C and 5E). Oviduct long, runs postero-sinistrally from the posterior margin of ovary (Fig. 1C and 5E). Mehlis’ gland small, well developed, surrounding oötype. Uterine seminal receptacle elongated, thick-walled (Fig. 1C and 5E). Laurer’s canal not observed. Uterus intercaecal, forming several transverse coils from uterine seminal receptacle to posterior margin of testicular field. Metraterm muscular, parallel to cirrus-sac (Fig. 1C). Vitellarium follicular. Vitelline fields mostly dorsal to caeca, from posterior level of nerve commissure to anterior margin of ovary on dextral side, and anterior to metraterm on sinistral side (Fig. 1A). Vitelline reservoir elongated, posterior to ovary. Vitelloduct connects to oviduct near oötype at sagittal axis (Fig. 1A-C, 5E). Fully developed eggs thin-shelled, sub-spherical to ellipsoidal.

Nerve commissure ventral, approximately at first sixth of oesophagus (Fig. 1A). Nerve cords extending anteriorly to posterior margin of mouth, indistinct posteriorly. Excretory vesicle Y-shaped; excretory pore subterminal (Fig. 1A and 3D).

#### 3.1.3 Remarks

Smith (1969) described *A. argentinensis* based on four specimens (holotype and three paratypes) from the bulbus arteriosus of *M. hubbsi* from off Argentinian coast. The illustration and description of Smith (1969) are incomplete and lack many structures, particularly those related to the reproductive organs. We re-examined the holotype (NHMUK, 1969.5.15.1), which was found to be considerably overstained (Fig. 6). The paratypes were not available for examination as they were kept in Smith’s collection. We also examined two mature, slightly contracted specimens from the bulbus arteriosus of *M. hubbsi* off the Falkland Islands (NHMUK, 1994.6.7.30-34). In addition, we examined extensive, newly well-fixed material, collected from both the heart/bulbus arteriosus and gill blood vessels of *M. hubbsi* from off northern Patagonia.

The metrical data for the type-, voucher-, and newly-collected material from our study provided additional measurements for the length of anterior and posterior caeca, uterine seminal receptacle and metraterm, the number and size of testes, the size of Mehlis’ gland, the width of cirrus-sac wall, and the distance from anterior body end to nerve commissure (Table 3). Our study extends the known range for the width of buccal capsule and ovary, and egg size. We also observed that the size of body, cirrus-sac, oesophagus and ovary, the number of spines, and the distance from posterior body end to genital pore in the material examined vary below the lower range given in the original description of the species (Table 3).

Our re-examination of the holotype and voucher specimens of *A. argentinensis* revealed discrepancies with the original description regarding the configuration of the buccal capsule and terminal genitalia. Smith (1969) did not report a buccal capsule in the anterior extremity of the alimentary canal. In his description, he mentioned that “the mouth is terminal and leads into the oesophagus”. However, we observed this structure during the re-examination of the holotype and vouchers (Fig. 5C). Furthermore, Smith (1969) described a genital atrium located approximately midway between the left lateral body margin and the dorsal midline, into which the cirrus and metraterm open. Our SEM micrographs indicate that the male and female genital pores open separately on the dorso-sinistral body surface of *A. argentinensis* (Fig. 3B). To our knowledge, the presence of a genital atrium has been reported in all species of *Aporocotyle* (Yamaguti, 1934, 1970; Williams, 1958; Smith, 1967; Ichihara, 1970; Holmes, 1971; Parukhin and Tkachuk, 1980; Thulin, 1980a; Fernández and Durán, 1985; Parukhin, 1985; Villalba and Fernández, 1986a, 1986b; Tantaleán and Martínez, 1990; Hernández-Orts et al., 2012, 2017; Santoro et al., 2015), and has been confirmed for *A. simplex* Odhner, 1900 by SEM (see figure 10 in Thulin, 1980b). The presence of a genital atrium in other species of *Aporocotyle*, and its value as a diagnostic morphological character to differentiate species should be confirmed based on light and scanning electron microscopy.

In addition to *A. argentinensis*, four species of *Aporocotyle* have been described from *Merluccius* spp.: *A. australis* ex Southern hake *M. australis* from off the Pacific coast of Chile, *A. margolisi* Smith, 1967 ex North Pacific hake *Merluccius productus* (Ayres) from off the Pacific coast of USA, *A. spinosicanalis* Williams, 1958 ex European hake *Merluccius merluccius* (L.) from off the northwest coast of the British Isles (Northeast Atlantic), and *A. wilhelmi* ex South Pacific hake *M. gayi* from off the Pacific coast of Chile (Hernández-Orts et al., 2017). These blood flukes possess similar morphology and have been distinguished based on relatively few morphological characters (e.g. Hernández-Orts et al., 2017). *Aporocotyle australis* is distinguished from *A. argentinensis* by the size of the body (5.4–8.0 × 1.2–1.9 mm *vs* 2.2–4.7 × 0.6–1.0 mm) and uterine seminal receptacle (246 *vs* 56–106 μm), length of oesophagus (1.5–2.2 *vs* 0.7–1.5 μm) posterior caeca (4417–4512 *vs* 1364–3312 μm) and metraterm (530 *vs* 97–375 μm), number of testes (56–71 *vs* 35–42 μm), distance from posterior body end to genital pore (1128 *vs* 480–1030 μm) and the ratios between the oesophagus and body length (1:3.7 *vs* 1:2.6–3.6) and the left anterior caecum and left posterior caecum (1:4.1 *vs* 1:1.7–3.7) (Table 3).

*Aporocotyle argentinensis* and *A. margolisi* are morphologically similar and have a close molecular relationship (see Hernández-Orts et al., 2017). However, these species can be differentiated by maximum body width (0.6–1.0 *vs* 1.1–1.4 mm), length of anterior and posterior caeca (636–1143 *vs* 1224–1719 μm and 1364–3221 *vs* 3444–5405 μm, respectively) and metraterm (97–375 *vs* 400–818 μm), and size of cirrus-sac (325–600 × 80–156 μm *vs* 510–1025 × 200–369 μm) and ovary (66–270 × 66–247 μm *vs* 280–329 × 150–421 μm).

*Aporocotyle spinosicanalis* can be distinguished from *A. argentinensis* particularly by the shape of the body margins which are curved ventrally to form a canal (see figure 3 in Smith, 1969). Also, these species differ by the number of testes (25–35 *vs* 35–42), disparate geographical distribution (northwest coast of the British Isles *vs* South America) (see Table 3) and by substantial genetic divergence (Hernández-Orts et al., 2017).

No significant morphological distinctions are apparent between *A. argentinensis* and *A. wilhelmi* (Table 3). In fact, Oliva et al. (2022) proposed that *A. wilhelmi* should be considered a junior synonym of *A. argentinensis* (see below). These species can be recognized exclusively by their distinct hake hosts (*M. hubbsi vs M. gayi*) and geographical distribution (Atlantic coast of Argentina *vs* Pacific coast of Chile and Peru).

Using the small subunit of the nuclear ribosomal rRNA (18S rRNA), 28S rRNA, and *cox*1 genes, Oliva et al. (2022) detected minimal genetic variation in *Aporocotyle* spp. infecting South American hakes. They therefore concluded that the three nominal species of *Aporocotyle* from South American hakes represent a single species which by the principle of priority correspond to *A. argentinensis*. Their results expanded the distribution of *A. argentinensis* to the Pacific coasts of Peru and Chile and reported new host records (*M. australis* and *M. gayi*) for this blood fluke. However, Oliva et al. (2022) did not provide morphological evidence to support their taxonomic arrangement, and the synonymization of *A. australis* and *A. wilhelmi* with *A. argentinensis* requires further evidence. In fact, Oliva et al. (2022) did not examine type-material of *Aporocotyle* spp. from South American hakes deposited in museum collections or link precisely identified, on morphological grounds, blood flukes from different hake species with their molecular data. Our morphological comparison suggests that *A. wilhelmi* might be a junior synonym of *A. argentinensis*. However, *A. argentinensis* and *A. australis* are morphologically distinct (Table 3). Therefore, examination of museum material, as well as the collection of new properly fixed specimens of *A. wilhelmi* and *A. australis* for combined morphological and molecular characterization, are needed to establish the taxonomic status of these species. Integrating molecular and morphological data is necessary to distinguish species that co-infect the same fish hosts, as shown for *Aporocotyle* species infecting ophidiiform fishes (Hernández-Orts et al., 2012; present study).

### 3.2 *Aporocotyle ymakara* Villalba & Fernández, 1986

#### 3.2.1 Taxonomic summary

*Type-host (same host in this study)*: Pink cusk-eel *Genypterus blacodes* (Förster) (Ophidiiformes: Ophidiidae).

*Type locality*: Golfo de Arauco (37°00′S, 73°20′W), Chile.

*Locality (this study)*: San Matías Gulf (an inlet of the Atlantic Ocean, 40°42′–40°45′S, 63°05′–65°10′W), Patagonia, Argentina (collected on 12.iv.2019).

*Other localities:* Off Central Patagonia (42°2′S, 7°19′ S–61°59′W, 64°25′W), Argentina (Hernández-Orts et al., 2012).

*Site in host:* Bulbus arteriosus (in this study), heart and gill blood vessels.

*Material studied:* New material: ex *G. blacodes*, gill blood vessels, San Matías Gulf, off Patagonia, Argentina, one voucher specimen: MHNG-PLAT-0160014.

*Representative DNA sequences:* PZ168273 (28S rDNA), PZ168639 (*cox*1 barcode region).

*Prevalence (this study)*: 11% (*n* = 9).

*Mean abundance (this study)*: 0.1 worms per fish.

*Mean intensity (this study)*: 1 worm per infected fish.

#### 3.2.2 Description

**Fig. 7:**
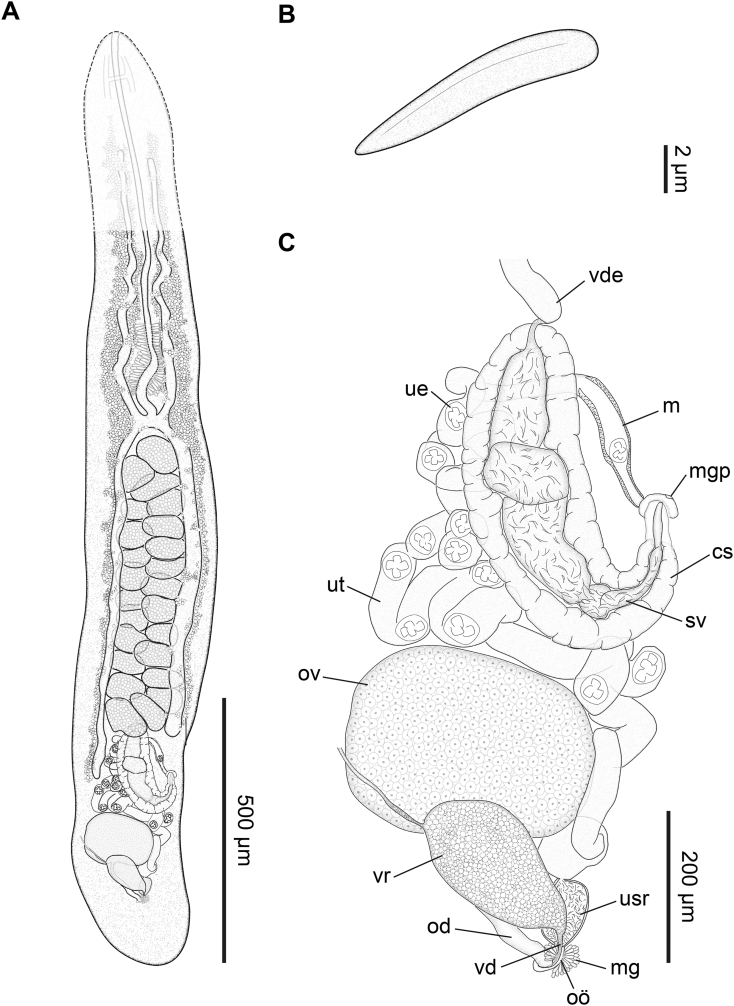
*Aporocotyle ymakara* Villalba & Fernández, 1986 (hologenophore) ex *Genypterus blacodes* (Förster). **A** Whole worm, ventral view. **B** Tegumental spine. **C** Reproductive organs, ventral view. *Abbreviations*: cs, cirrus-sac; mgp, genital pore; m, metraterm; mg, Mehlis’ gland; od, oviduct; oö, oötype; ov, ovary; sv, seminal vesicle; ue, uterine egg; usr, uterine seminal receptacle; ut, uterus; vd, vitelloduct; vr, vitelline reservoir.

[Based on a single gravid hologenophore; morphometric data in Table 4; Fig. 5, Fig. 7.] Body lanceolate, with blunt anterior end and slightly pointed posterior end (Fig. 5B and 7A); maximum width approximately at midbody. Tegument spines (Fig. 7B) in clusters of approximately 20 spines each, in ventro-lateral fields (Fig. 5G-I), slightly extending dorsally; ventral clusters are distributed along entire body length, never joining at sagittal axis, but reaching closer at anterior half body, occupying, approximately 2/3 distance between body margin to caeca; spines less abundant at posterior half body. Oesophagus surrounded by glands at its posterior half until caecal bifurcation (Fig. 7A). Intestine H-shaped. Right anterior caecum slightly longer than left anterior caecum. Right posterior caecum longer than left posterior caecum. Right posterior caecum ending approximately at cirrus-sac level (Fig. 7A). Left posterior caecum ending before posterior margin of testes field (Fig. 7A).

**Table 4:** Comparative measurements for *Aporocotyle ymakara* Villalba & Fernández, 1986 ex *Genypterus blacodes* (Förster) from off the Pacific and Atlantic coasts of South America. Measurements are in micrometres unless otherwise indicated.

Source	Villalba and Fernández (1986a)	Hernández-Orts et al. (2012)	Present study
Host	*G. blacodes*	*G. blacodes*	*G. blacodes*
Locality	Southeast Pacific, Golfo de Arauco, off Chile	Southwest Atlantic, off Central Patagonia, Argentina	San Matias Gulf, off Northern Patagonia, Argentina
Site in host	Bulbus arteriosus and gill blood vessels	Bulbus arteriosus	Gill blood vessels
Feature	Range (*n* = 40)	Range (*n* = 1)	Range (*n* = 1)
BL × BW (mm)	1.6–2.7 × 0.3–0.4	1.7 × 0.4	1.8 × 0.3
BCL × BCW	38 × 21	–	–
SL	10	18	8–11
NSC	–	20	13–14
OL (mm)	0.8–1.2	0.7	0.8
MEPC	Cirrus-sac level^a^	Cirrus-sac level	Cirrus-sac level
RACL	760^a^	333	556
LACL	730^a^	310	524
RPCL	520–1060	674	702
LPCL	470–910	508	635
NT	18–21	18	24
TLA × TWA	30–61 × 36–80	23–64 × 39–81	59–103 × 38–60
CSL × CSW	40–130 × 120–240	263 × 70	233 × 64
CSWW	–	19^a^	16
DPGP	424^a^	450^a^	412
OVL × OVW	40–120 × 90–150	121 × 144	90 × 131
MGL × MGW	–	27 × 48^a^	20 × 20
USRL	52^a^	43^a^	35
ML	108^a^	79	71
EL × EW	–	13–19 × 17–23	13–14 × 9–12
DANC	98^a^	87^a^	86
*Ratios*			
OL/BL	1:2.1–2.6	1:2.6^a^	1:2.4
RACL/RPCL	1:3.5	1:2.0^a^	1:1.3
LACL/LPCL	1:2.8	1:1.6^a^	1:1.3
RPCL/BL	1:2.6^a^	1:2.5^a^	1:2.6
LPCL/BL	1:2.6^a^	1:3.3^a^	1:2.9

a Obtained from the published figure.

Testes irregularly shaped (Fig. 5B and 7A), intercaecal, between intestinal bifurcation and anterior margin of reproductive organs. Vas deferens extending from posterior border of testicular field. Cirrus-sac claviform, with a thick muscular wall, directing sinistrally, ending in genital atrium (Fig. 7C). Seminal vesicle elongate, slightly sinuous, in middle of cirrus-sac, prostatic cells not observed (Fig. 7C). Genital atrium dorso-sinistral (Fig. 7C).

Ovary central, slightly dextral, sub-ellipsoidal, in posterior 1/8 of body (Fig. 7A). Oviduct running posteriorly from posterior margin of ovary, sinistrally oriented (Fig. 7C). Oötype surrounded by Mehlis’ gland, connecting to uterine seminal receptacle. Laurer’s canal not observed. Uterus extending from uterine seminal receptacle and coiling from posterior ovary margin to level of anterior cirrus-sac end, curving dorso-sinistrally (Fig. 7C). Metraterm thin walled, parallel to cirrus-sac (Fig. 7C). Vitelline follicles small. Vitelline fields branched, extending to posterior end of right posterior caecum, and, on sinistral side, slightly before the end of left posterior caecum. Vitelline reservoir posterior to ovary (Fig. 7C). Vitelloduct posterior to vitelline reservoir, short, connecting ventrally to oviduct. Eggs thin-shelled, irregular to ellipsoidal shaped. Excretory pore not observed.

#### 3.2.3 Remarks

This species was described by Villalba and Fernández (1986b) for specimens collected from pink cusk-eels *G. blacodes* from off the Arauco Gulf in Chile. It was distinguished from other species of *Aporocotyle* by its small body size, oesophagus to body length ratio, anterior caeca to posterior caeca ratio and the number of testes. *Aporocotyle ymakara* was reported and illustrated later from pink cusk-eels *G. blacodes* from the central Patagonia shelf in Argentina by Hernández-Orts et al. (2012). Our newly-collected specimen extends the lower range for the size of Mehlis’ gland, the length of spines, uterine seminal receptacle and metraterm, the width of cirrus-sac and eggs, the distance from posterior body end to genital pore and the anterior caeca to posterior caeca ratio. In addition, our study extends the maximum range for testis width and the number of testes (Table 4). The position of the cirrus-sac in the new specimen overlapped dorsally with the posterior-most testis. In previous descriptions, the cirrus-sac was distant to or abutting, but not overlapping, the posterior-most testis or slightly more distally (Villalba and Fernández, 1986b; Hernández-Orts et al., 2012).

### 3.3 *Aporocotyle mariachristinae* Hernández-Orts, Alama-Bermejo, Carrillo, García, Crespo, Raga & Montero, 2012

#### 3.3.1 Taxonomic summary

*Type-host (same host in this study)*: Pink cusk-eel *Genypterus blacodes* (Förster) (Ophidiiformes: Ophidiidae).

*Type-locality*: Off North Patagonia (42°45′S–42°59′S, 61°09′W–62°58′W); and Central Patagonia (42°2′S–47°19′S, 61°59′W–64°25′W), Argentina.

*Locality (this study)*: San Matías Gulf (an inlet of the Atlantic Ocean, 40°42′–40°45′S, 63°05′–65°10′W), Patagonia, Argentina (collected on 05.xii.2018).

*Other localities*: Off Puerto Montt (41°28′11″S, 72°56′41″W), Los Lagos Region, Chile (Cifuentes Riquelme, 2015).

*Site in host*: Bulbus arteriosus, ventral aorta and gill blood vessels.

*Material studied*: New material: ex *G. blacodes*, gill blood vessels, San Matías Gulf, Patagonia, Argentina. Both specimens were used for SEM.

*Prevalence (this study)*: 11% (*n* = 9 fish).

*Mean abundance (this study)*: 0.2 worms per fish.

*Mean intensity (this study)*: 2 worms per infected fish.

#### 3.3.2 Description

**Fig. 8:**
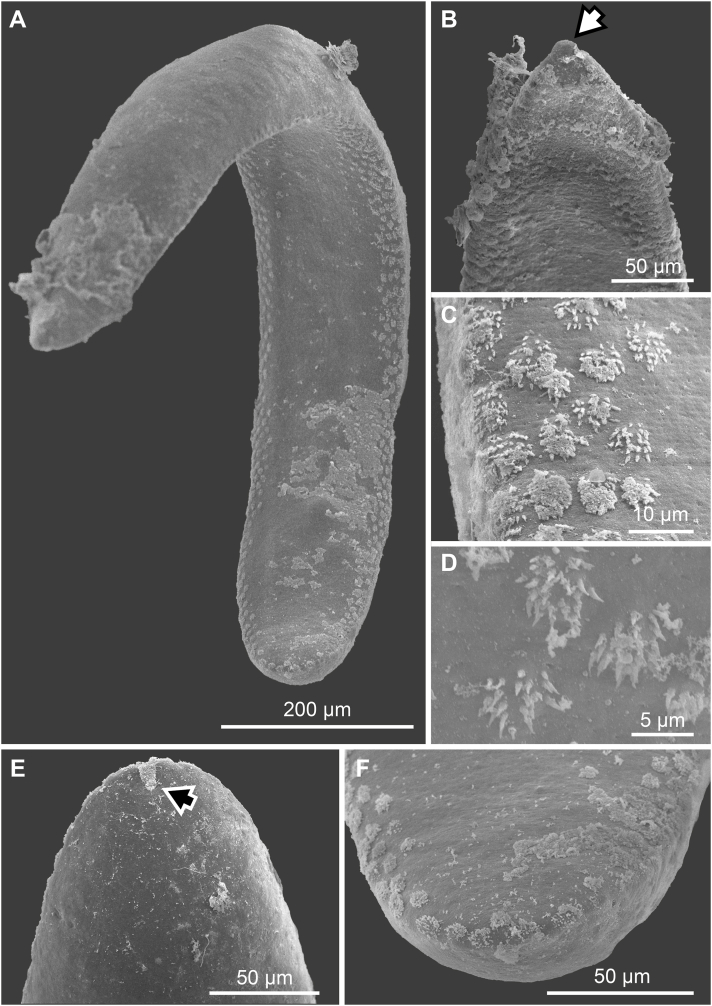
Scanning electron micrographs of *Aporocotyle mariachristinae* Hernández-Orts, Alama-Bermejo, Carrillo, García, Crespo, Raga & Montero, 2012 ex *Genypterus blacodes* (Förster). **A** Whole worm, dorso-ventral view. **B** Anterior region, ventral view (*arrow* points to mouth). **C** Ventro-lateral body area showing the distribution of clusters of spines. **D** Clusters of spines. **E** Posterior part of the worm, dorsal view (*arrow* points to excretory pore). **F** Posterior part of the worm, ventral view.

[Based on two specimens examined by SEM; Fig. 8.] Body narrowly lanceolate (Fig. 8A), with blunt anterior end (Fig. 8B) and slightly pointed posterior end (Fig. 8E and F). Oral sucker absent. Mouth subterminal (Fig. 8B). Tegumental spines short and pointed, clusters of 20 to 31 spines each (Fig. 8C and D). Cluster of spines distributed along ventro-lateral margins of body (Fig. 8A), never joining at sagittal axis but reaching closer near posterior margin of mouth (Fig. 8B); clusters of spines less abundant on posterior third of body on ventral side (Fig. 8A). Clusters of spines absent on dorso-lateral margins of body (Fig. 8A-E). Excretory pore subterminal, on dorsal surface of body (Fig. 8E).

#### 3.3.3 Remarks

Before fixation in hot 4% formaldehyde, the two worms examined in this study were identified as *A. mariachristinae* based on their large body size and the characteristic asymmetrical extension of the posterior caeca. In this species, the right posterior caecum is longer, ending between the mid-level of the ovary and the posterior end of the body, whereas the left posterior caecum is shorter, ending between the mid-level of the cirrus-sac and the posterior end of the reproductive organs (see Hernández-Orts et al., 2012).

*Aporocotyle mariachristinae* was originally described from specimens collected in the bulbus arteriosus and ventral aorta of pink cusk-eels *G. blacodes* from northern and central Patagonia, Argentina. This species was later reported in pink cusk-eels *G. blacodes* from Puerto Montt in Chile (Cifuentes Riquelme, 2015). The present study reports the gill blood vessels as a new microhabitat for this species.

The distribution of spines along the body has been shown to be a useful distinguishing feature in *Aporocotyle* (see Hernández-Orts et al., 2012). Clusters of spines restricted to the ventro-lateral margins of the body have been reported only in five of the 18 described species of *Aporocotyle* (i.e. *A. garciai*, *A. keli*, *A. kuri*, *A. ymakara*, and *A. mariachristinae*), all of which infect three species of *Genypterus* (Hernández-Orts et al., 2012). Parukhin and Tkachuk (1980) described the body of *A. smithi* Parukhin & Tkachuk, 1980 from the kingklip *Genypterus capensis* (Smith) as being entirely covered by clusters of tegumental spines. However, their figure 2 shows the clusters distributed along the ventro-lateral margins of the body. Therefore, examination of both type- and newly collected material of *A. smithi* is required to clarify the distribution of spines along the body in this species.

### 3.4 Molecular analyses

Altogether, 13 new sequences were generated in this study. We produced sequences for three markers for *A. argentinensis*, four for the 28S rRNA gene (1286–1307 nt), two for the barcode region of the *cox*1 gene (786 nt), and five for the middle region of the *cox*1 gene (675 nt). In addition, we sequenced two genes for *A. ymakara*, the 28S rRNA gene (1273 nt) and the *cox*1 barcode region (425 nt).

**Fig. 9:**
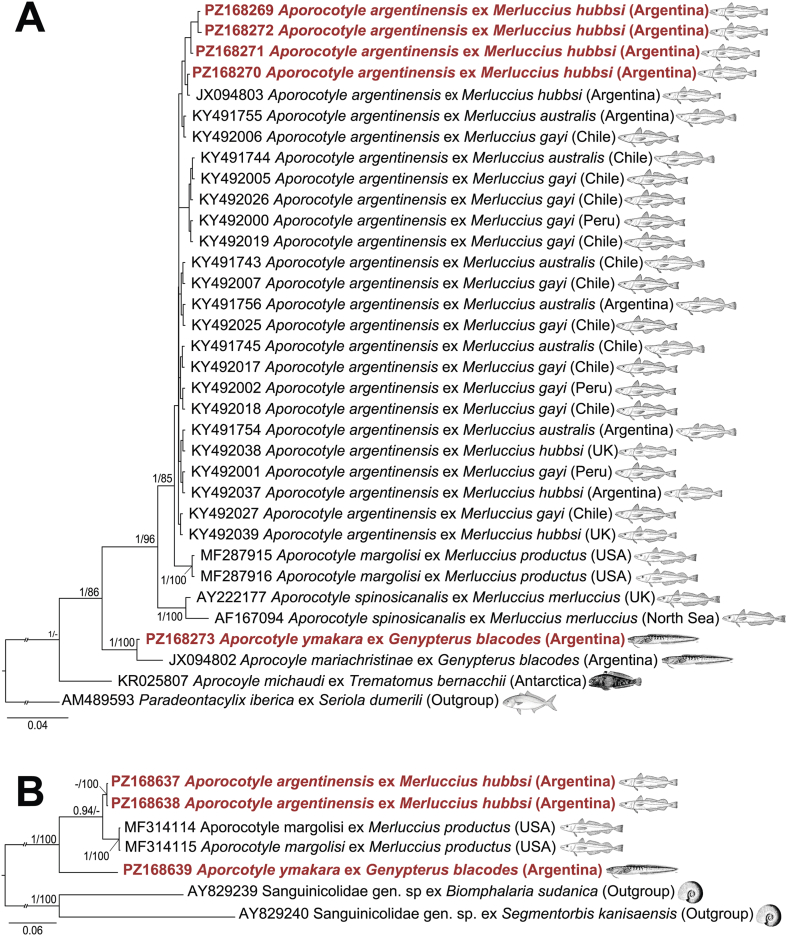
Bayesian inference (BI) and ML trees derived from the 28S rRNA gene (**A**) and the *cox*1 barcode region (**B**) sequences for species of *Aporocotyle*, with nodal support given by posterior probabilities (> 0.95 shown only) followed by bootstrap values (> 70% shown only). The newly generated sequences are indicated in red. The scale-bar indicates the expected number of substitutions per site.

The BI and ML analyses of the 28S rDNA dataset (6 taxa, 35 sequences, 913 nt) produced identical tree topologies. The new sequences of *A. argentinensis* resolved in a poorly-supported clade together with selected sequences for isolates identified as *A. argentinensis* from three hake species (*M. hubbsi*, *M. gayi* and *M. australis*) from off Argentina, Chile, Peru and the Falkland Islands (Oliva et al., 2022) retrieved from the GenBank database. The *A. argentinensis* clade appeared as sister to a clade formed by two sequences of *A. margolisi*, with strong posterior probability support (Fig. 9A). The two 28S rDNA sequences for *A. spinosicanalis* (GenBank: AY222177 and AF167094) published by Olson et al. (2003) and Snyder and Loker (2000) were placed with strong support as sister to the clade formed by *A. argentinensis* and *A. margolisi* (Fig. 9A). Genetic distances within the *A. argentinensis* clade ranged from 0 to 0.3%, and between *A. argentinensis* and *A. margolisi* was 0.7%. Genetic divergence between the two sequences of *A. spinosicanalis* was 1.0%.

**Fig. 10:**
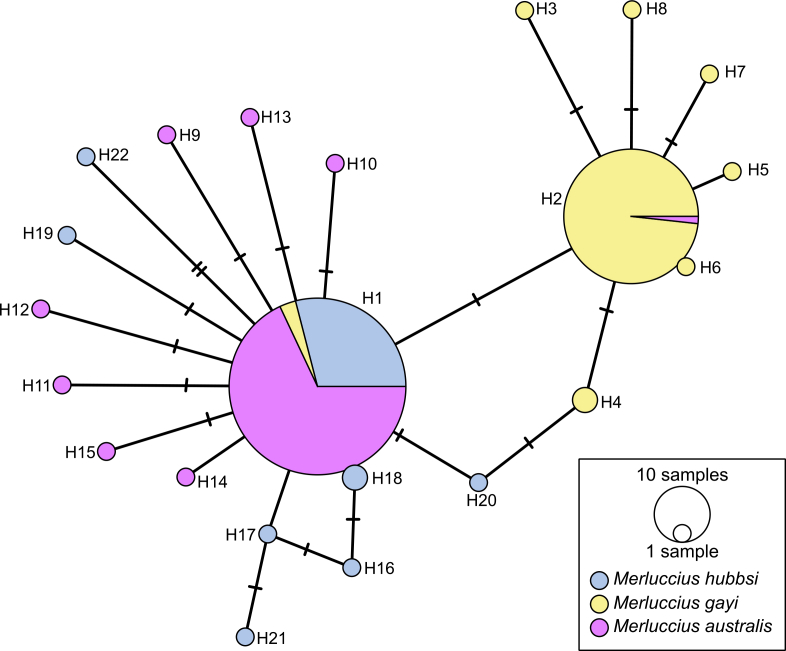
Haplotype network of *Aporocotyle argentinensis* Smith, 1969 based on partial *cox*1 gene sequences (middle region) from three species of *Merluccius* of the southeastern Pacific and southwestern Atlantic coasts of South America (*n* = 180 sequences, 675 nt, Table 2). The hatch marks on the connecting lines indicate the number of the mutational steps between the haplotypes. The size of the pie chart is proportional to the number of isolates sharing the same haplotype.

The novel sequence of the isolate identified as *A. ymakara* ex *G. blacodes* from San Matías Gulf, formed a strongly supported clade with the sequence of *A. mariachristinae* ex *G. blacodes* from off Patagonia, Argentina (Fig. 9A). Genetic distance between *A. ymakara* and *A. mariachristinae* was 1.1%. The two species of *Aporocotyle* from *G. blacodes* formed a strongly-supported clade sister to the clade formed by the three species of *Aporocotyle* from *Merluccius* spp. In our study, *A. michaudi* Santoro, Cipriani, Pankov & Lawton, 2015 was basal to all other *Aporocotyle* species (Fig. 9A).

The phylogenetic analysis for the barcode region of *cox*1 dataset (5 taxa, 7 sequences, 792 nt) placed our two novel sequences of *A. argentinensis* as sister to the sequences of *A. margolisi* (Fig. 9B). The novel sequence of the isolate identified as *A. ymakara* formed a strongly supported lineage sister to the clade consisting of *A. argentinensis* + *A. margolisi* (Fig. 9B). The two novel *cox*1 sequences for *A. argentinensis* were identical. Genetic distances between *A. argentinensis* and *A. margolisi* was 2.3%. Uncorrected p-distance between *A. ymakara* and *A. argentinensis* was 11.3%, and between *A. ymakara* and *A. margolisi* was 12.7%.

The middle region of the *cox*1 gene dataset for *A. argentinensis* included 180 sequences (675 nt) generated from isolates collected from *M. hubbsi*, *M. australis* and *M. gayi* from the Pacific (off Peru and Chile) and Atlantic (off Argentina and Falkland Islands) of South America (Oliva et al., 2022) (Table 2). Intraspecific sequence variability in this dataset ranged from 0 to 0.6%. A haplotype network was built with this *cox*1 dataset, revealing 21 distinct haplotypes (Fig. 10). The topology of our haplotype network was similar to that provided by Oliva et al. (2022), showing the presence of two major haplogroups. The first major haplogroup (H1) corresponded mostly to specimens collected from *M. gayi* off Peru and Chile. The second major haplogroup (H2) includes specimens collected mainly from *M. australis* and *M. hubbsi* off Chile, Argentina and the Falkland Islands. The new *cox*1 sequences for *A. argentinensis* from San Matías Gulf were placed on haplotype H1 (Fig. 10). Sequences showed a moderate haplotype diversity (*Hd* = 0.590) and low nucleotide diversity (*π* = 0.0011). The average number of nucleotide differences was also low (*κ* = 0.741).

## 4 Discussion

The genus *Aporocotyle* was proposed by Odhner (1900) to accommodate *A. simplex* infecting the European plaice *Pleuronectes platessa* L. (Pleuronectidae) from off Gullmarsfjorden on the Swedish west coast. The genus comprises 18 species infecting 10 fish families belonging to the Gadiformes (i.e. Gadidae, Macrouridae and Merlucciidae), Ophidiiformes (i.e. Ophidiidae), Perciformes (i.e. Nototheniidae, Psychrolutidae and Sebastidae), Pleuronectiformes (i.e. Bothidae and Pleuronectidae) and Scombriformes (i.e. Gempylidae) (Hernández-Orts et al., 2012; Santoro et al., 2015). Most species of *Aporocotyle* are distributed widely but the major number of species, i.e. *A. argentinensis*, *A. australis*, *A. garciai*, *A. keli*, *A. kuri*, *A. mariachristinae*, *A. wilhelmi* and *A. ymakara*, are found along the coasts of South America. *Aporocotyle simplex* has been reported in northern cold-temperate marine waters, i.e. Arctic, North Atlantic, including the North Sea and Baltic region and North Pacific/Far East; however, some records, particularly from the Far East, have been questioned (Gibson, 1996). *Aporocotyle spinosicanalis* has been reported in marine waters of the northeastern Atlantic and the Mediterranean Sea (Ferrer-Maza et al., 2014). The other nominal species are found in Antarctic waters (*A. michaudi*), the Northeast Pacific coast of the USA (*A. macfarlani* Holmes, 1971 and *A. margolisi*), the Sea of Japan (*A. orientalis* Yamaguti, 1934 and *A. theragrae* Ichihara, 1970), the Indian Ocean (*A. nototheniae* Parukhin, 1985 and *A. smithi*), and off Hawaii in the Pacific (*A. pacifica* Yamaguti, 1970) (see Hernández-Orts et al., 2012; Santoro et al., 2015). To the best of our knowledge, *A. garciai* is the only species reported in both hemispheres: the Pacific coast of Japan and the Southeast Pacific off the Peruvian coast (Tantaleán and Martínez, 1990; Kamegai et al., 2002), but the record of this species in Japan needs to be confirmed.

Twelve species of *Aporocotyle* are apparently strict specialists, each parasitizing a single host species (Hernández-Orts et al., 2012; Santoro et al., 2015). As far as we know, six species occur in multiple host species: *A. simplex* have been reported in 16 marine fish taxa of five families belonging to the Gadiformes, Pleuronectiformes and Perciformes (Thulin, 1980a, 1991; Shvetsova, 2005); *A. theragrae* in three gadiform species, i.e. the Alaska pollock *Gadus chalcogrammus* Pallas, the Pacific cod *Gadus macrocephalus* Tilesius, and the North Pacific hake *M. productus* (Ichihara, 1970; Shvetsova, 2005); *A. wilhelmi* in two hake species, i.e. the South Pacific hake *M. gayi* and the Peruvian hake *Merluccius peruanus* Ginsburg (Villalba and Fernández, 1986a; Martínez et al., 2013; Chero et al., 2014); *A. garciai* in ophidiiform fishes, i.e. *Genypterus* sp. and the armoured cusk *Hoplobrotula armata* (Temminck and Schlegel) (Tantaleán and Martínez, 1990; Kamegai et al., 2002); *A. orientalis* in two psychrolutid fishes, i.e. *Cottunculus* sp. and *Malacocottus gibber* Sakamoto (Yamaguti, 1934; Kuramochi, 2014), and *A. argentinensis* which was reported in three species of *Merluccius* from off South America (Oliva et al., 2022). However, the true host specificity of many *Aporocotyle* spp., including those described from South America and the Far Eastern seas, remains unclear and requires critical reassessment.

The diversity of *Aporocotyle* parasitizing hakes *Merluccius* spp. off South America remains unresolved, as the validity of two species has been questioned by Oliva et al. (2022). Based on the current morphological and molecular evidence, *A. wilhelmi* is likely a junior synonym of *A. argentinensis*. In contrast, the validity of *A. australis* is more controversial, as numerous morphological characters, previously used to differentiate species, clearly distinguish it from *A. argentinensis* (Table 3). Traditionally, the most used morphological characters to distinguish species of *Aporocotyle* are the body shape, the distribution and arrangement of tegumental spines along the body, the oesophagus/body length ratio, the anterior caeca to posterior caeca ratio, the posterior caeca to body length ratio, the number of testes, the shape of the cirrus-sac and ovary, and the maximum extension of posterior caeca (see below). Although intraspecific variability of some of these morphological characters has not been explored in detail, available evidence suggests that they could differ between individuals collected from different host species (Thulin, 1980a) or localities (Hernández-Orts et al., 2017). The validity of the synonymy of *A. wilhelmi* and *A. australis* with *A. argentinensis* needs to be justified based on molecular data linked to adequate morphological descriptions of well-fixed specimens and museum material. Resolving this taxonomic puzzle could provide a useful model for exploring intraspecific variability in fish blood flukes associated with different hosts and geographical regions.

Six species of *Aporocotyle* have been reported infecting cusk-eels *Genypterus* spp. (Ophidiidae) from the coasts of South America (*A. keli*, *A. kuri, A. garciai*, *A. ymakara* and *A. mariachristinae*) and South Africa (*A. smithi*) (Parukhin and Tkachuk, 1980; Villalba and Fernández, 1986b; Tantaleán and Martínez, 1990; Hernández-Orts et al., 2012). Cifuentes Riquelme (2015) provided the first phylogenetic assessment of aporocotylids infecting South American pink cusk-eels *Genypterus* spp. using 18S rRNA and 28S rRNA genes. Their analyses suggested that *A. keli* and *A. kuri* represent a single species (with priority for *A. kuri*) and reported *A. mariachristinae* and an unidentified *Aporocotyle* species from the pink cusk-eel *G. blacodes*. The sequences generated by Cifuentes Riquelme (2015) are not available in GenBank and could not be included in our analyses. However, the 28S tree in Cifuentes Riquelme (2015) placed the unidentified *Aporocotyle* as sister to *A. mariachristinae* with strong bootstrap support. In our analyses, *A. ymakara* was recovered as sister to *A. mariachristinae* in a well-supported clade (Fig. 9A), producing a topology identical to that reported in figure 6 by Cifuentes Riquelme (2015). This suggests that the unidentified *Aporocotyle* reported by Cifuentes Riquelme (2015) corresponds to *A. ymakara* as characterised in our study. The phylogenetic relationships of *A. garciai* and *A. smithi* with other *Aporocotyle* species infecting cusk-eels *Genypterus* spp. remain unresolved, as no molecular sequence data are currently available for them.

Aporocotylids generally have few morphological characteristics which reliably distinguish species. Morphological plasticity of characters currently employed to circumscribe and classify species, such as the body size, the oesophagus length, and the ovary dimensions, are known to differ between populations of *A. margolisi* parasitizing the North Pacific hake *M. productus* (Hernández-Orts et al., 2017). The size and number of testes have frequently been considered important in the identification of *Aporocotyle* species (e.g. Hernández-Orts et al., 2012); however, these characters may be influenced by degeneration of the worm’s reproductive organs (Thulin, 1980a). In this study, we noted the absence of a genital atrium in *A. argentinensis* (Fig. 3B), a structure that has been described in all *Aporocotyle* species*.* The presence of a genital atrium, and its utility as a reliable morphological trait for delimiting species, should be examined as additional material from other species becomes available.

The maximum extension of posterior caeca has been shown to be a useful distinguishing feature in *Aporocotyle* (Villalba and Fernández, 1986b; Hernández-Orts et al., 2012). Posterior caeca terminating near the posterior body end (Fig. 1A) have been reported in most *Aporocotyle* species parasitizing fishes belonging to the Pleuronectiformes, Gadiformes, Perciformes and Scombriformes. In five species that parasitize Ophidiiformes, i.e. *A. garciai*, *A. keli*, *A. kuri*, *A. smithi*, and *A. ymakara*, the posterior caeca are shorter, terminating between the posterior margin of testes and the mid-level of cirrus-sac, never reaching the ovary (Fig. 7A). Only in *A. mariachristinae*, which parasitize Ophidiformes, the posterior caeca are markedly asymmetrical, with the right posterior caecum always longer than left posterior caecum (Hernández-Orts et al., 2012). In this species, the right posterior caecum terminates from mid-level of ovary to near the posterior body end, and the left posterior caecum ends from mid-level of cirrus-sac to posterior to reproductive organs (see figure 1 in Hernández-Orts et al., 2012).

The distribution and arrangement of tegumental spines along the body is one of the most important features in the taxonomy of *Aporocotyle*. In our study, the distribution of tegumental spines were examined using for the first time SEM for *A. argentinensis* and *A. mariachristinae*. In *A. argentinensis*, the tegumental spines are arranged in clusters (Fig. 4C), which are distributed along the lateral body margins, joining at sagittal axis from posterior end of the mouth to approximately caeca bifurcation on the ventral side (Fig. 4A), and restricted to the lateral body margins on the dorsal side (Fig. 3A and B). The arrangement of these spines was observed by light microscopy for type- and voucher material of *A. spinosicanalis*, *A. margolisi* and *A. wilhelmi* by Hernández-Orts et al. (2012, 2017) and was described for *A. australis* and *A. theragrae* (Ichihara, 1970; Fernández and Durán, 1985). In contrast, clusters of tegumental spines are exclusively distributed along ventro-lateral margins of the body in *A. mariachristinae* (Fig. 8A), but also in *A. garciai*, *A. keli*, *A. kuri, A. macfarlani*, *A. simplex* and *A. ymakara* (Holmes, 1971; Thulin, 1980b; Tantaleán and Martínez, 1990; Hernández-Orts et al., 2012). Interestingly, in *A. simplex,* six rows of minute tegumental spines were described at the anterior body end (Thulin, 1980a, 1980b). Four species, namely *A. nototheniae*, *A. orientalis*, *A. pacifica* and *A. smithi*, were described with clusters of tegumental spines covering the entire body (Parukhin, 1985; Yamaguti, 1934, 1970; Parukhin and Tkachuk, 1980), but this distribution and arrangement should be confirmed with advanced imaging. Finally, *A. michaudi* is the only described species lacking clusters of spines on its body surface (Santoro et al., 2015). In this species, the body is entirely covered by single conical spines, with seven concentric rows of single spines near its anterior body end. The arrangement and distribution of tegumental spines are taxonomically important structures not easily seen by light microscopy. Therefore, SEM should be considered a standard technique for morphological studies of *Aporocotyle*, as it provides three-dimensional images of taxonomically important structures useful for future comparative anatomical studies.

Sequence data are available for six species of the Aporocotylidae: three infecting fishes of the Merlucciidae, i.e. *A. argentinensis*, *A. margolisi* and *A. spinosicanalis*; two in fishes of the Ophidiidae, i.e. *A. ymakara* and *A. mariachristinae*; and one in fishes of the Nototheniidae, i.e. *A. michaudi*. In our 28S rDNA phylogenetic analysis, the three species infecting merluccids formed a clade that was the sister group of the two species infecting ophidiids. The topology retrieved in this tree, resolved *A. michaudi* as the sister species of all other species of *Aporocotyle*. Of interest will be the inclusion of the type-species, i.e. *A. simplex* as it possesses morphological characters of both *A. michaudi* (e.g. rows of minute spines near the anterior body end) and more derived species (e.g. clusters of spines). In addition, a more intensive sampling effort with broader geographical coverage, particularly targeting species infecting Gadiformes, Perciformes, and Scombriformes in the North Pacific and Indian Oceans, is necessary to better understand the diversity and systematics of *Aporocotyle*.

In our 28S rDNA dataset, the two isolates identified as *A. spinosicanalis* from *M. merluccius*, from the North Sea (GenBank: AF167094; Snyder and Loker, 2000) and from the UK (GenBank: AY222177; Olson et al., 2003), exhibited a high level of intraspecific variation (1.0%). This value slightly exceeds the genetic divergence observed between closely related species of *Aporocotyle* in our dataset. Although the observed variation may be attributable to base-calling errors, the possibility that these sequences represent distinct taxa or misidentified material cannot be excluded. The taxonomy and systematics of *A. spinosicanalis* therefore require further scrutiny through an integrative approach from worms collected from the European hake *M. merluccius* across different localities in the North and the Mediterranean Seas.

The haplotype network reconstruction confirmed the genetic similarity of *A. argentinensis* sequences from the San Matías Gulf with those from other regions off Argentina, Chile, and Peru (Oliva et al., 2022). Our novel sequences contribute to the mapping of *A. argentinensis* populations in South America, revealing relatively low genetic diversity based on our haplotype analysis of this blood fluke species across its hake hosts (*M. australis*, *M. hubbsi*, and *M. gayi*) in the southwestern Atlantic and southeastern Pacific. Although overall genetic variability was less prominent in our network, two major haplogroups were identified (Fig. 10). This pattern might indicate a low intraspecific differentiation within *A. argentinensis* or the presence of two closely related taxa: one infecting *M. gayi* from off Peru and Chile, and another corresponding to *A. argentinensis* infecting *M. australis* and *M. hubbsi* from off Chile, Argentina, and the Falkland Islands. Unfortunately, the published sequences lacked a comprehensive morphological assessment (Oliva et al., 2022), leaving the taxonomic identity of the haplogroup formed by sequences from isolates collected from *M. gayi* unresolved.

Among *Aporocotyle* spp., only the life cycle of *A. simplex* has been elucidated, with the terebellid polychaete *Artacama proboscidea* Malmgren acting as the intermediate host (Køie, 1982). Additionally, the terebellid *Lanassa nordenskioldi* Malmgren has been reported as an intermediate host for an unidentified species of *Aporocotyle* (Køie and Petersen, 1988). The life cycle of the South American species of *Aporocotyle* remains unknown. However, the high infection levels of *A. argentinensis* in the Argentine hake *M. hubbsi* may be a consequence of the abundance of its intermediate host and the subsequent successful infection. More than ten families of marine polychaetes, including aphroditids, eunicids, arabellids, terebellids, etc., have been reported as accompanying fauna in shrimp and hake catches from the Patagonian shelf in Argentina (Roux et al., 2005; Bovcon et al., 2013). However, records of deep-water polychaetes from San Matías Gulf remain scarce, and, to date, no terebellid species have been reported from this inlet area of the Atlantic Ocean.

## 5 Conclusions

Blood flukes represent a neglected and understudied group of digeneans infecting marine fishes in the southwestern Atlantic. Our survey of fish blood flukes from off northern Patagonia revealed three species of *Aporocotyle* from the circulatory system of the Argentine hake *M. hubbsi* and the pink cusk-eel *G. blacodes*. Our ultrastructural analyses using confocal and scanning electron microscopy provided detailed observations of taxonomically important structures, including the arrangement and distribution of tegumental spines and the presence of two distinct genital pores in *A. argentinensis*. The occurrence of either a genital atrium or separate genital pores has traditionally been regarded as an important genus-level diagnostic character in aporocotylids. The fact that species of *Aporocotyle* might have both across the genus has significant taxonomic importance, not only for this genus but also for other genera. Future attempts to verify whether other species had one or two genital pores are necessary to elucidate the importance of these characters for the classification of these trematodes. Our molecular study expanded the 28S rDNA and *cox*1 sequence databases with 13 sequences for two species of *Aporocotyle* from gadiform and ophidiiform fishes. Our phylogenetic study also detected minimal genetic diversity for aporocotylids recovered from three hake species (*M. hubbsi*, *M. australis*, and *M. gayi*) from off the coasts of South America. These results suggest the presence of a single species of *Aporocotyle* infecting South American hakes, which should correspond to *A. argentinensis*. We highlight that an integrative taxonomic approach is required to validate the synonymy of *A. wilhelmi* and *A. australis* with *A. argentinensis.* Finally, future molecular studies targeting species of *Aporocotyle* infecting Gadiformes, Perciformes, and Scombriformes in the North Pacific and Indian Oceans are essential to improve our understanding of the evolutionary relationships and global diversity of this genus.

## Ethical approval

Approval from a research ethics committee was not required for this study, as fish blood flukes were obtained from dead fish purchased from local fishermen.

## CRediT authorship contribution statement

**Marta Valmaseda-Angulo:** Writing - original draft, Writing - review & editing, Investigation, Validation, Data curation. **Gema Alama-Bermejo:** Methodology, Investigation, Writing - review & editing. **Francisco E. Montero:** Methodology, Investigation, Writing - review & editing. **David I. Hernández-Mena:** Investigation, Formal analysis, Writing - review & editing. **Jesús S. Hernández-Orts:** Conceptualization, Investigation, Formal analysis, Data curation, Writing - original draft, Writing - review & editing, Resources, Funding acquisition.

## Funding

This study was financially supported by the 10.13039/501100001824Czech Science Foundation (project 23-05733S) and PAPIIT (Biodiversidad de helmintos de peces dulceacuícolas en peligro de extinción en México; project IA206125).

## Declaration of competing interests

The authors declare that they have no known competing financial interests or personal relationships that could have appeared to influence the work reported in this paper.

## Data Availability

All data generated or analyzed during this study are included in this published article. The voucher specimens are deposited at the Helminthological Collection of the Museo de La Plata, Buenos Aires, Argentina (HCMLP-He 8349 and 8350), the Natural History Museum, Geneva, Switzerland (MHNG-PLAT-0160014), the Colección Nacional de Helmintos, Mexico City, Mexico (CNHE 11086, 11087), and the Natural History Museum, London (NHM, 2025.10.31.1-17). The newly generated sequences are deposited in the GenBank database under the accession numbers PZ168269-PZ168273, PZ168637-PZ168639, and PZ168640-PZ168644.

## References

[bib1] Bandelt H.J., Forster P., Röhl A. (1999). Median-joining networks for inferring intraspecific phylogenies. Mol. Biol. Evol..

[bib2] Bovcon N.D., Góngora M.E., Marinao C., González-Zevallos D. (2013). Composición de las capturas y descartes generados en la pesca de merluza común *Merluccius hubbsi* y langostino patagónico *Pleoticus muelleri*: un caso de estudio en la flota fresquera de altura del Golfo San Jorge, Chubut, Argentina. Rev. Biol. Mar. Oceanogr..

[bib3] Braicovich P.E., Etchegoin J.A., Timi J.T., Sardella N.H. (2006). A new species of *Cardicola* Short, 1953 (Digenea: Sanguinicolidae) parasitizing the Brazilian flathead, *Percophis brasiliensis* Quoy et Gaimard 1824, from the coasts of Mar del Plata, Argentina. Parasitol. Int..

[bib4] Brant S.V., Morgan J.A., Mkoji G.M., Snyder S.D., Rajapakse R.P., Loker E.S. (2006). An approach to revealing blood fluke life cycles, taxonomy, and diversity: provision of key reference data including DNA sequence from single life cycle stages. J. Parasitol..

[bib5] Chero J.D., Cruces C., Oliver J.I., Saez Flores G., Alvariño L., Rodríguez C. (2014). Parasitological indices of the Peruvian hake *Merluccius gayi peruanus* (Ginsburg, 1954) (Perciformes: Merlucciidae) acquired at the fishing terminal of Ventanilla, Callao, Peru. Neotrop. Helminthol..

[bib6] Cifuentes Riquelme M. (2015). Filogenia de *Aporocotyle* spp. (Digenea) en sus hospedadores del género *Merluccius* y *Genypterus*, en Chile. Bachelor’s Thesis, Valdivia, Universidad Austral de Chile, Chile.

[bib7] Fernández J., Durán L. (1985). *Aporocotyle australis* n. sp. (Digenea: Sanguinicolidae), parásito de *Merluccius australis* (Hutton 1872) en Chile y su relación con la filogenia de *Aporocotyle* Odhner, 1900 en *Merluccius* spp. Rev. Chil. Hist. Nat..

[bib8] Ferrer-Maza D., Lloret J., Muñoz M., Faliex E., Vila S., Sasal P. (2014). Parasitism, condition and reproduction of the European hake (*Merluccius merluccius*) in the northwestern Mediterranean Sea. ICES J. Mar. Sci..

[bib9] George-Nascimento M. (1996). Populations and assemblages of parasites in hake, *Merluccius gayi*, from the southeastern Pacific Ocean: stock implications. J. Fish. Biol..

[bib10] Gibson D.I., Margolis L., Kabata Z. (1996).

[bib11] González-Poblete L., Saavedra J.C., Céspedes R., Canales C.M. (2022). Parasites of *Merluccius australis* as biological tags to determine the hake ecological stocks in the sea channels of Chilean Patagonia. Estuar. Coast Shelf Sci..

[bib12] Hernández-Mena D.I., Lynggaard C., Mendoza-Garfias B., Pérez Ponce de León G.P. (2016). A new species of *Auriculostoma* (Trematoda: Allocreadiidae) from the intestine of *Brycon guatemalensis* (Characiformes: Bryconidae) from the Usumacinta River Basin, Mexico, based on morphology and 28S rDNA sequences, with a key to species of the genus. Zootaxa.

[bib13] Hernández-Orts J.S., Alama-Bermejo G., Carrillo J.M., García N.A., Crespo E.A., Raga J.A. (2012). *Aporocotyle mariachristinae* n. sp., and *A. ymakara* Villalba & Fernández, 1986 (Digenea: Aporocotylidae) of the pink cusk-eel, *Genypterus blacodes* (Ophidiiformes: ophidiidae) from Patagonia, Argentina. Parasite.

[bib14] Hernández-Orts J.S., Hernández-Mena D.I., Alama-Bermejo G., Kuchta R., Jacobson K.C. (2017). Morphological and molecular characterisation of *Aporocotyle margolisi* Smith, 1967 (Digenea: Aporocotylidae) from the North Pacific hake *Merluccius productus* (Ayres) (Gadiformes: Merlucciidae) off Oregon, USA. Syst. Parasitol..

[bib15] Holmes J.C. (1971). Two new sanguinicolid blood flukes (Digenea) from scorpaenid rockfishes (Perciformes) of the Pacific coast of North America. J. Parasitol..

[bib16] Ichihara A. (1970). A new blood fluke *Aporocotyle theragrae* n. sp. (Digenetic trematode: *Aporocotyle*) from a marine fish, *Theragra chalcograma*. Res. Bull. Meguro Parasitol. Mus..

[bib17] Kalyaanamoorthy S., Minh B.Q., Wong T.K.F., von Haeseler A., Jermiin L.S. (2017). ModelFinder: fast model selection for accurate phylogenetic estimates. Nat. Methods.

[bib18] Kamegai S., Machida M., Kuramochi T. (2002). Two blood flukes from deep-sea fishes of Suruga Bay, Japan. Bull. Nat. Sci. Mus. Tokyo, Ser. A.

[bib19] Katoh K., Standley D.M. (2013). MAFFT multiple sequence alignment software version 7: improvements in performance and usability. Mol. Biol. Evol..

[bib20] Køie M. (1982). The redia, cercaria and early stages of *Aporocotyle simplex* Odhner, 1900 (Sanguinicolidae) - a digenetic trematode which has a polychaete annelid as the only intermediate host. Ophelia.

[bib21] Køie M., Petersen M.E. (1988). A new annelid intermediate host (*Lanassa nordenskioeldi* Malmgren, 1866) (Polychaeta: Terebellidae) for *Aporocotyle* sp. and a new final host family (Pisces: Bothidae) for *Aporocotyle simplex* Odhner, 1900 (Digenea: Sanguinicolidae). J. Parasitol..

[bib22] Kumar S., Stecher G., Suleski M., Sanderford M., Sharma S., Tamura K. (2024). Mega 12: Molecular Evolutionary Genetic Analysis version 12 for adaptive and green computing. Mol. Biol. Evol..

[bib23] Kuramochi T. (2014). Digenean trematodes of deep-sea fishes from the Sea of Japan. Natl. Mus. Nat. Sci. Monogr..

[bib24] MacKenzie K., Longshaw M. (1995). Parasites of the hakes *Merluccius australis* and *M. hubbsi* in the waters around the Falkland Islands, southern Chile, and Argentina, with an assessment of their potential value as biological tags. Can. J. Fish. Aquat. Sci..

[bib25] Martínez R.R., Tantaleán M., Mondragón A.M. (2013). Primer registro de *Macrophyllida antarctica* (Monogenea, Trochopodinae) y *Aporocotyle wilhelmi* (Digenea, Aporocotylidae) en peces de la costa peruana. Peru J. Parasitol..

[bib26] Miura O., Kuris A.M., Torchin M.E., Hechinger R.F., Dunham E.J., Chiba S. (2005). Molecular-genetic analyses reveal cryptic species of trematodes in the intertidal gastropod, *Batillaria cumingi* (Crosse). Int. J. Parasitol..

[bib27] Moszczynska A., Locke S.A., McLaughlin J.D., Marcogliese D.J., Crease T.J. (2009). Development of primers for the mitochondrial cytochrome *c* oxidase I gene in digenetic trematodes (Platyhelminthes) illustrates the challenge of barcoding parasitic helminths. Mol. Ecol. Resour..

[bib28] Nadler S.A., Hudspeth D.S.S. (1998). Ribosomal DNA and phylogeny of the Ascaridoidea (Nemata: Secernentea): implications for morphological evolution and classification. Mol. Phylogenet. Evol..

[bib29] Odhner T. (1900). *Aporocotyle simplex* n. g., n. sp., ein neuer Typus von ektoparasitischen Trematoden. Centr. Bakteriol. Parasitenkd. Infektionskr..

[bib31] Oliva M.E., Cárdenas L., Valdivia I.M., Bruning P., Figueroa-Fabrega L., Escribano R. (2022). Spatial pattern of genetic diversity in the blood fluke *Aporocotyle argentinensis* (Digenea, Aporocotylidae) from South American hakes (Pisces: Merluccidae). Diversity.

[bib30] Oliva M.E., Ballón I. (2002). Metazoan parasites of the Chilean hake *Merluccius gayi gayi* as a tool for stock discrimination. Fish. Res..

[bib32] Olson P.D., Cribb T.H., Tkach V.V., Bray R.A., Littlewood D.T. (2003). Phylogeny and classification of the Digenea (Platyhelminthes: Trematoda). Int. J. Parasitol..

[bib33] Parukhin A.M. (1985). New trematode species from commercial fish of the Indian Ocean. Nauchn. Dokl. Vyssh. Shk. Biol. Nauki.

[bib34] Parukhin A.M., Tkachuk L.P. (1980). New species of trematodes from fish in the Indian Ocean. Nauchn. Dokl. Vyssh. Shk. Biol. Nauki.

[bib35] Pleijel F., Jondelius U., Norlinder E., Nygren A., Oxelman B., Schander C. (2018). Phylogenies without roots? A plea for the use of vouchers in molecular phylogenetic studies. Mol. Phylogenet. Evol..

[bib36] Rambaut A. (2009). FigTree. Tree figure drawing tool. http://tree.bio.ed.ac.uk/software/figtre.

[bib37] Repullés-Albelda A., Montero F.E., Holzer A.S., Ogawa K., Hutson K.S., Raga J.A. (2008). Speciation of the *Paradeontacylix* spp. (Sanguinicolidae) of *Seriola dumerili*. Two new species of the genus *Paradeontacylix* from the Mediterranean. Parasitol. Int..

[bib38] Ronquist F., Teslenko M., van der Mark P., Ayres D.L., Darling A., Höhna S. (2012). MrBayes 3.2: efficient Bayesian phylogenetic inference and model choice across a large model space. Syst. Biol..

[bib39] Roux A., Bremec C., Schejter L., Giberto D. (2005). Benthic invertebrates by-catch of demersal fisheries: a comparison between Subantarctic and Antarctic shelf waters (45°S–57°S). Ber. Polar Meeresforsch..

[bib40] Rozas J., Ferrer-Mata A., Sánchez-DelBarrio J.C., Guirao-Rico S., Librado P., Ramos-Onsins S.E. (2017). DnaSP 6: DNA sequence polymorphism analysis of large data sets. Mol. Biol. Evol..

[bib41] Santoro M., Cipriani P., Pankov P., Lawton S.P. (2015). *Aporocotyle michaudi* n. sp. (Digenea: Aporocotylidae) from the emerald rock cod, *Trematomus bernacchii* (Teleostei: Perciformes) in Antarctica. Parasitol. Int..

[bib42] Sardella N.H., Timi J.T. (1996). Parasite communities of *Merluccius hubbsi* from the Argentinian-Uruguayan common fishing zone. Fish. Res..

[bib43] Schindelin J., Arganda-Carreras I., Frise E., Kaynig V., Longair M., Pietzsch T. (2012). Fiji: an open-source platform for biological-image analysis. Nat. Methods.

[bib44] Shvetsova L.S. (2005). [Trematodes of the genus *Aporocotyle* (Sanguinicolata: Aporocotylidae) from fish in Far Eastern seas]. Parazitologiya.

[bib45] Smith J.W. (1967). *Aporocotyle margolisi* n. sp. (Digenea: Aporocotylidae) from *Merluccius productus*. J. Fish. Res. Board Can..

[bib46] Smith J.W. (1969). On *Aporocotyle argentinensis* n. sp. (Digenea: Sanguinicolidae) from *Merluccius hubbsi*, and the phylogeny of *Aporocotyle* Odhner, 1900 in hake. J. Helminthol..

[bib47] Snyder S.D., Loker E.S. (2000). Evolutionary relationships among the Schistosomatidae (Platyhelminthes: Digenea) and an Asian origin for *Schistosoma*. J. Parasitol..

[bib48] Stock S.P., Campbell J.F., Nadler S.A. (2001). Phylogeny of *Steinernema* Travassos, 1927 (Cephalobina: Steinernematidae) inferred from ribosomal DNA sequences and morphological characters. J. Parasitol..

[bib49] Swofford D.L. (2002).

[bib50] Tantaleán M., Martínez R. (1990). *Aporocotyle garciai* n. sp. (Digenea: Sanguinicolidae), parásito de *Genypterus* sp. de la costa peruana. Parasitol. Día.

[bib53] Thulin J. (1991). *Aporocotyle simplex*, a blood fluke in flatfish. ICES Identif. Leafl. Dis. Parasites fish Shellfish Rep..

[bib51] Thulin J. (1980). A redescription of the fish blood-fluke *Aporocotyle simplex* Odhner, 1900 (Digenea, Sanguinicolidae) with comments on its biology. Sarsia.

[bib52] Thulin J. (1980). Scanning electron microscope observations of *Aporocotyle simplex* Odhner, 1900 (Digenea: Sanguinicolidae). Z. Parasitenkd..

[bib54] Van Steenkiste N., Locke S.A., Castelin M., Marcogliese D.J., Abbott C.L. (2015). New primers for DNA barcoding of digeneans and cestodes (Platyhelminthes). Mol. Ecol. Resour..

[bib55] Villalba C., Fernández J. (1986). Dos nuevas especies de trematodos parásitos de peces marinos en Chile. Parasitol. Día.

[bib56] Villalba C., Fernández J. (1986). Tres nuevas especies de *Aporocotyle* Odhner, 1900 (Digenea: Sanguinicolidae) parasitas de *Genypterus* spp. en Chile (Pisces: Ophidiidae). Rev. Biol. Mar. Valparaíso.

[bib57] Villora-Montero M., Hernandez-Orts J.S., Palacios-Abella J.F., Montero F.E. (2025). *Yamagu-Tips*, digital scientific drawings of helminths and other microscopic organisms. Zootaxa.

[bib58] Warren M.B., Bullard S.A. (2023). Systematic revision of the fish blood flukes with diagnoses of Chimaerohemecidae Yamaguti, 1971, Acipensericolidae n. fam., Sanguinicolidae Poche, 1926, Elopicolidae n. fam., and Aporocotylidae Odhner, 1912. J. Parasitol..

[bib59] Williams H.H. (1958). The anatomy of *Aporocotyle spinosicanalis* sp. nov. (Trematoda: Digenea) from *Merluccius merluccius* (L.). J. Nat. Hist. Ser..

[bib60] Wong T.K.F., Ly-Trong N., Ren H., Baños H., Roger A.J., Susko E. (2026). IQ-TREE 3: phylogenomic inference software using complex evolutionary models. Mol. Biol. Evol..

[bib61] Xia X. (2018). DAMBE7: new and improved tools for data analysis in molecular biology and evolution. Mol. Biol. Evol..

[bib63] Xia X., Xie Z., Salemi M., Chen L., Wang Y. (2003). An index of substitution saturation and its application. Mol. Phylogenet. Evol..

[bib62] Xia X., Lemey P., Lemey P., Salemi M., Vandamm A.M. (2009). The Phylogenetic Handbook: a Practical Approach to DNA and Protein Phylogeny.

[bib64] Yamaguti S. (1934). Studies on the helminth fauna of Japan. Part 2. Trematodes of fishes. I. Jpn. J. Zool..

[bib65] Yamaguti S. (1970).

